# Interdisciplinary research approach based on a mixed-methods design to explore patient altruism at the end of life: a study protocol

**DOI:** 10.1136/bmjopen-2024-085632

**Published:** 2024-05-10

**Authors:** Mathieu Bernard, Claudia Gamondi, Anca-Cristina Sterie, Philip J Larkin, Ralf J Jox, Gian Domenico Borasio

**Affiliations:** 1 Palliative and Supportive Care Service, Chair of Palliative Psychology, Lausanne University Hospital and University of Lausanne, Lausanne, Vaud, Switzerland; 2 Palliative and Supportive Care Service, Lausanne University Hospital and University of Lausanne, Lausanne, Vaud, Switzerland; 3 Palliative and Supportive Care Service and Service of Geriatric Medicine and Geriatric Rehabilitation, Chair of Geriatric Palliative Care and Chair of Palliative Psychology, Lausanne University Hospital and University of Lausanne, Lausanne, Vaud, Switzerland; 4 Palliative and Supportive Care Service, Chair of Palliative Care Nursing, Lausanne University Hospital and University of Lausanne, Lausanne, Vaud, Switzerland; 5 Palliative and Supportive Care Service and Service of Geriatric Medicine and Geriatric Rehabilitation, Chair of Geriatric Palliative Care, Lausanne University Hospital and University of Lausanne, Lausanne, Vaud, Switzerland

**Keywords:** PALLIATIVE CARE, Adult palliative care, SOCIAL MEDICINE

## Abstract

**Introduction:**

In the end of life context, patients are often seen as somewhat passive recipients of care provided by health professionals and relatives, with little opportunity to be perceived as autonomous and active agents. Since studies show a very high prevalence of altruistic dispositions in palliative care patients, we strive to investigate the concept of patient altruism in a set of six interdisciplinary studies by considering three settings: (1) in the general palliative context—by studying to what extent patient altruism is associated with essential psychological outcomes of palliative care (subproject 1a), how altruism is understood by patients (subproject 1b) and how altruism expressed by patients is experienced by palliative care nurses (subproject 1c); (2) in two concrete decision-making contexts—advance care planning (subproject 2a) and assisted suicide (subproject 2b); and (3) through verbal and non-verbal patient communication in palliative care settings (subproject 3).

**Methods and analysis:**

Subproject 1a: a cross-sectional study using validated and standardised questionnaires. Subprojects 1b and 1c: a constructivist grounded theory method aiming at developing a novel theory from semistructured interviews in both patients and nurses. Subproject 2a: a thematic analysis based on (1) audio-recordings of advance care planning encounters and (2) follow-up semidirective interviews with patients and their relatives. Subproject 2b: a qualitative study based on thematic analysis of interviews with patients actively pursuing assisted suicide and one of their relatives.Subproject 3: a conversation analysis based on audio and video-recorded interactions in two settings: (1) palliative inpatient unit and (2) advance care planning discussions.

**Ethics and dissemination:**

The study project was approved by the Ethics Committees of the Canton of Vaud, Bern and Ticino (no: 2023-00088). In addition to participation in national and international conferences, each project will be the subject of two scientific publications in peer-reviewed journals. Additional publications will be realised according to result triangulation between projects. A symposium opened to professionals, patients and the public will be organised in Switzerland at the end of the project.

**STRENGTHS AND LIMITATIONS OF THIS STUDY**

- We propose a set of multidisciplinary, complementary and interlinked studies to explore altruism in palliative care in three different settings.
- A complex concept such as patient altruism can only be addressed if several disciplines and methods are used.
- Each subproject is conceived to explore a specific facet of patient altruism at the end of life and relies on a specific methodology of analysis corresponding to their research questions.
- A joint phase of data and results triangulation between the subprojects will allow exchanges between teams on results and new interpretations of phenomena.
- As a limitation, patients and the public were not involved in the design of the study.

## Introduction

### Palliative care challenges

In palliative care, the psychological domain is an essential component of patient care. Previous research in the field has mostly focused on improving the pharmacological and psychological treatments for the most frequent psychopathologies, that is, anxiety, depression and adjustment disorders.^1 2^ Less is known concerning the factors that foster psychological well-being, autonomy and self-determination, thus improving quality of life. This approach, which has been termed ‘positive psychology’,^3^ parallels the resource-oriented, health-promoting approach known as salutogenesis. In recent years, palliative care patients have been considered as active participants in their own dying, but only concerning specific and well-defined aspects of the end of life, namely, the so-called decisions concerning end of life (ie, advance directives, withholding and withdrawing life-sustaining treatment, and requests for hastened death/assisted suicide where legally possible). In all other aspects concerning the last phase of life, patients are rather regarded as passive recipients of care, with only little opportunity to be perceived as ‘agents’ who act, give and contribute something by and of themselves. One possible way of fostering patient autonomy and well-being at the end of life is to consider the patient in line with the concept of ‘patient empowerment’. While this paradigm is trending in many medical disciplines,^4^ it has had little impact in the field of palliative care so far, with the exception of those situations where decisions need to be made. It is precisely for this reason that we place the concept of patient altruism, as a form of patient agency, at the centre of this project.

Nowadays, the concept of altruism is used in various disciplines such as psychology, philosophy, economics, sociology and evolutionary biology.^5 6^ Numerous authors tend to apply this concept to any prosocial behaviour or action carried out on a voluntary basis and aiming to benefit the society at large, specific individuals or a group of individuals.^5 7–9^ While altruistic behaviour is primarily explained as an individual constitutive characteristic,^10–13^ this behaviour has to be understood by considering its sources of motivation (benevolence,^11^ empathy,^14^ reward,^15^ norms^16^ and anger^5 17^). Social norms and interaction rules (social responsibility, group gain, reciprocity, negotiated rules) have also been identified as determinants of altruistic behaviours.^18^ To adequately address this multilevel concept (personality, motivations, social norms), an interdisciplinary approach appears necessary, which is also underscored by the different disciplines involved in this project (humanities, social sciences, medical sciences and nursing).

Although palliative care addresses all people suffering from a life-limiting illness, most palliative care patients who benefit from it are close to end of their life and are of older age. It is well established that older people report higher altruism levels than younger people.^19^ This is in agreement with life span developmental theories that have shown a change in motivation orientation during the last phase of existence, which promotes a sense of realisation and meaning in life.^20^ While meaning in life tends to increase with age,^21 22^ the sources of meaning change: Sparrow and Spaniol showed that, with increasing age, intrinsic values (such as authenticity, intimacy, spirituality but also altruism) are prioritised over extrinsic goals, such as achievement, competence and power.^23^ Other authors mention a shift towards meaningful social goals focusing on others, especially close ones, after the recognition that time is becoming more limited as age increases.^24^ Vollhardt suggests that altruism may also be a consequence of suffering after adverse life events, such as a life-threatening illness.^25 26^ In line with these findings, Fegg *et al* showed that palliative care patients consistently reported higher self-transcendent and altruistic values as compared with healthy adults.^27^ In addition, palliative care patients cite the social dimension as a source of meaning in life more often than the general population.^28^

Since the fragility of patients at the end of life often reduces their capacity to undertake concrete altruistic actions by themselves, we need to broaden our understanding of altruism by also taking into account the attitudes and values at the origin of altruistic behaviours. In the theoretical model of basic human values developed by Schwartz and Cieciuch, the values of ‘benevolence’ and ‘universalism’ (‘self-transcendence’) refer to concern for others’ welfare and represent an internal motivation promoting social relations.^29 30^ Since there is currently no clear definition or conceptual framework of altruism that has been applied to palliative care patients, we propose defining altruism in this context as: ‘a personal and intentional interest in improving the well-being and welfare of others—at an individual, group or societal level’. By emphasising the notion of ‘interest’, we aim to include the different levels of manifestations of altruism that might be relevant to palliative care, including the (advance) decisions made by patients and the interactions upon which these decisions are based.

We therefore propose a set of multidisciplinary, complementary and interlinked studies to explore altruism in palliative care in three different settings:

- The first level refers to patient altruism in the general context of palliative care:
  - We first aim to study to what extent altruism, considered in terms of prosocial behaviour and self-transcendent value, is associated with important outcomes and indicators of palliative care (subproject 1a).
  - We then aim to explore (1) how altruism is defined and understood by the palliative care patients themselves (subproject 1b) and (2) how altruism from patients is experienced by palliative care nurses, the professionals who are closest to patients in clinical practice, and how the expression of altruism by patients and families towards nurses influences professional meaning and fulfilment (subproject 1c).
- The second level considers how altruism might be expressed and realised through specific decisions between patients and their social environment by considering two specific decision-making contexts of high importance for palliative care: advance care planning (ACP, subproject 2a) and assisted suicide (subproject 2b). Both represent profound existential expressions of autonomy, which are closely related to the patients’ values and attitudes.
- The third level concerns how altruism might be expressed in social interactions (both verbal or non-verbal), and how the interaction itself might have an altruistic dimension. We consider moments in which patients are involved in interaction by their own volition, and in which such interaction might bring a potential benefit to the other (subproject 3).

This project is anchored in an interdisciplinary effort bringing together the expertise of several researchers in palliative healthcare. They are therefore representative of the interdisciplinary nature of palliative care, as defined by the WHO.^31^ Mathieu Bernard, head of subprojects 1a and 1b, is a psychologist; Phil J Larkin, head of subproject 1c, is a nurse; Ralf Jox, head of subproject 2a, and Claudia Gamondi, head of subproject 2b, are physicians, as Gian Domenico Borasio, project coordinator. Finally, Anca-Cristina Sterie, head of subproject 3, is a sociologist specialised in interactions in the medical setting.

The repercussions of this project could be manifold: first, it would allow designing and testing an altruism-based intervention for palliative care patients that could represent an important new step in the development of efficient, resource-oriented palliative care. Second, such interventions would have the potential to restore dignity and autonomy for patients in the last phase of life by allowing them, if they so wish, to assume a more active role. Third, the expression of patient altruism towards family members and healthcare professionals could also profoundly affect the latter two: it could diminish their distress and ease their grieving, improve relationships and may be a role model for themselves to become more altruistic.

## Methods

### Subproject 1a

A cross-sectional study using validated and standardised questionnaires will be conducted in both the French-speaking and German-speaking parts of Switzerland (two university hospitals and five palliative care centres), and two palliative care centres in the Italian-speaking part of Switzerland.

#### Inclusion criteria

- 18 years or older.
- Treated by one of the palliative care teams.
- <6 months’ life expectancy according to the treating physician.
- Medically stable state or when their state has improved.

#### Exclusion criteria

- Evidence of psychiatric and cognitive symptoms that might significantly alter the decision-making capacity.
- Insufficient knowledge of the local language.

#### Questionnaires and procedure

For the purpose of the study, we will use:

- The Prosocialness Scale for Adults.^32^
- The benevolence (four items) and universalism (six items) subscales of the 40-item Portrait Values Questionnaire.^30^
- Quality of life will be measured with the McGill Quality of Life Scale Revised version.^33^
- Psychological distress will be measured with the Hospital Anxiety and Depression Scale.^34^
- Patients’ feeling of being a burden to their caregivers will be assessed with the Self-Perceived Burden Scale.^35^
- Meaning in life will be assessed with the Meaning in Life Questionnaire.^36^
- The will to live intensity will be measured with a single-item Numerical Rating Scale.^37^

These questionnaires were chosen because they measure dimensions that have been identified as psychological determinants of quality of life in palliative care. All new patients admitted in the palliative care centres participating in the study and who fulfil the inclusion and exclusion criteria as assessed by the referring physician will be asked by a research assistant to participate in the study as soon as an appropriate acute symptom control has been reached. Written informed consent will be obtained.

Validated translations or cross-cultural adaptations of the questionnaires will be used in face-to-face interviews. In addition, sociodemographic variables and medical data (principal diagnosis, comorbidities, performance status) will be obtained. Finally, questions using Numerical Rating Scales (0–10) will assess the level of stress and the potential for personal development induced by the questionnaires. Such Numerical Rating Scales will also be used to assess (1) to what extent altruism is an important concept in the end of life context, and (2) to what extent patients feel frustration not to be able to express altruism towards others.

#### Data analysis strategy

Rates will be reported for the recruitment information. Descriptive statistics will be calculated for sociodemographic variables and all outcomes. Missing outcome data will be treated as mentioned in the scoring manuals. Distribution of each outcome measure will be analysed using normality tests and other indicators of distribution (box-plots, skewness and kurtosis estimations). Associations between outcome variables will be assessed using Pearson or Spearman correlations according to distributions. Univariate and multiple linear regressions will be performed in order to determine whether altruism can be considered as a significant explanatory variable (in addition to psychological distress, desire to live, feeling of being a burden and meaning in life) for quality of life. We will use an alpha threshold of <0.05 as a criterion for rejecting the null hypothesis. All models will be controlled for sociodemographic characteristics.

#### Sample size

Multiple linear regressions represent the crucial elements for assessing the number of participants to be included to ensure sufficient power. According to Howell,^38^ we aim to recruit 15 patients per explanatory variable, that is, 120 patients with complete data in this study (50 in the French-speaking and German-speaking parts and 20 in the Italian-speaking part, according to population representativeness). Estimating an exclusion rate of 60% and a refusal rate of 50% based on previous studies in the same context,^39–41^ 600 patients in total will need to be screened for study eligibility (250 in the French-speaking and German-speaking parts and 100 in the Italian-speaking part).

### Subproject 1b

#### Procedure and sample size

A subsample of participants from project 1a will be invited to participate in subproject 1b. Agreement to participate will be sought at the end of project 1a data collection. We estimate a maximum sample of 45 participants (approximately 15 per linguistic region), depending on data saturation (REF) and the need to explore items in greater or lesser depth. Therefore, the final sample may not be determined until the end of the study. We will aim to maximise the sample variation by ensuring diversity across gender, age, setting of care, illness type (eg, cancer vs non-cancer) and also by considering the score on the two questionnaires assessing altruism.

A constructivist grounded theory method will be adopted for projects 1b and 1c.^42^ In this qualitative method, the researcher inductively analyses individual and collective actions as well as social and psychological processes, approaching the data with no preconceived ideas or hypotheses, accepting that there may be multiple perspectives and acknowledging the importance of their own place in constructing those perspectives with participants. Data collection and analysis are undertaken in parallel and decisions on sampling are revised as the project develops, a process referred to as ‘theoretical sampling’.

An initial interview guide will be developed based on topics derived from data collected in subproject 1a and from research questions of subproject 1b. Semistructured interviews will be conducted by a researcher trained in qualitative methodology and grounded theory data analysis, supported by qualitative research experts. The semistructured approach enables the interviewer to vary the wording and sequence of the questions and pursue leads provided by the participants, while being respectful of the burden of interviewing a frail and potentially vulnerable population.

The interview guide will be developed by the research team, comprising native speakers in French, German, Italian and English. The team will meet after the first five interviews in each region to discuss emerging themes and agree if new directions in terms of revised questions are needed. Theoretical saturation will be determined first in each region and later agreed through consensus with the wider research team.

#### Data analysis

All interviews will be digitally recorded and transcribed in the language of origin. To account for language and cultural differences, interpretation and analysis of the data will be carried out by research collaborators in each region, trained in qualitative methodology and supported by the local research partners. Data will be entered into MAXQDA software to assist analysis.

In accordance with constructivist grounded theory methods, a broad pool of codes will be obtained during an open (initial) coding using interview data, notes and memos. In a second phase of axial (focused) coding, all individual codes will be sorted and resorted as concepts and common themes are developed.^42^ In the final step (theoretical coding), overarching themes will be grouped into more refined categories and central concepts, again referring to wider data sources (notes, memos, etc). Each coding step will be conducted in the individual language for the region and data shared through regular collaborative meetings to ensure accuracy and quality. An iterative discussion process will be engaged throughout the study, in order to reach a substantial agreement between researchers in terms of major themes and any cultural or regional difference identified. The final themes will be translated into English as the common language for the presentation of data and subjected to a second-level inductive analysis and comparison with the host language for accuracy in terms of conceptual equivalence. The emerging theory will be co-constructed by the research team.

### Subproject 1c

#### Procedure and sample size

We first begin with a conceptual analysis of altruism to determine its antecedents, attributes and relationships with palliative care nursing. Conceptual analyses are of particular benefit where a concept requires refinement within a specific discipline or context.^43^ This will also inform the development of the interview guide and the outcome can be refined during interviews with the nursing participants.

Following the constructivist grounded theory principles outlined in subproject 1b, 30 semistructured in-depth interviews with nurses working in palliative care nursing practice will be undertaken. Participants will be accessed through the same university hospitals and palliative care centres collaborating for subprojects 1a and 1b (French, German and Italian). Interviews will provide concrete examples of the nurses’ experience of altruism in practice and address the meaning and impact of altruism for the palliative care nurse practitioner.

#### Inclusion criteria for nurses

- Working at specialist or generalist level within an agreed palliative care setting.
- Having worked in a full-time or part-time capacity for at least 6 months to be able to reflect and discuss their professional experience and offer concrete examples from practice.
- Willing to be interviewed and recorded.
- Able to converse in either French, German, Italian or English.

Since the division between specialist and generalist palliative care nursing roles is ill-defined, interviews with a broader nursing sample will help to understand the broader facilitators and barriers to altruism and to seek ulterior expressions and meanings of altruism within a wider professional nursing cohort. 10 interviews per linguistic region are planned.

#### Data analysis

The process of data analysis described in subproject 1b will also be applied to subproject 1c for symmetry and completeness of data across subprojects 1b and 1c. It will then afford opportunity to consider both datasets for similarities and disparities in the understanding and expression of altruism.

### Subproject 2a

In order to address the aims of subproject 2a, a qualitative design will be used with three types of data: (1) audio or video-recorded routine ACP discussions between a facilitator, a patient and their relative(s) if applicable; (2) semistructured follow-up interviews of the persons having participated in these discussions.

#### Inclusion criteria for patients

- 18 years or older.
- Receiving general or specialised palliative care.

#### Inclusion criteria for relatives

- Being nominated and recruited by the patient.
- 18 years or older.
- Capable to participate in an interview in French or German.

#### Inclusion criteria for ACP facilitators

- Acting as a facilitator of ACP discussions with the patient (and his or her relatives).
- Being trained as an ACP facilitator.

#### Exclusion criteria for patients and relatives

- Evidence of psychiatric or cognitive symptoms significantly altering the decision-making capacity.

#### Procedure and sample size

We will follow a sampling strategy combining cluster sampling and convenience sampling. The cluster sampling will be oriented towards variation regarding gender, age groups and health status of the patient participants (oncological, neurological and cardiorespiratory illnesses). The convenience part of the sampling relates to the fact that we will recruit in structured and professionally facilitated ACP activities in the university hospitals of both the French and German parts of Switzerland, and in two palliative care centres of the Italian part of Switzerland. It is planned to conduct 9–12 qualitative interviews with ACP facilitators (3–4 per language region), and to register 24 audio-recorded routine ACP discussions (8 in each language area), as well as 24 follow-up interviews with patients and 24 follow-up interviews with relatives having participated in the ACP discussions.

#### Data collection

The ACP discussions will be audio or video-recorded. Audio-recordings will be transcribed verbatim. The transcripts will then be coded by the investigator and analysed by the researcher.

The semistructured interviews will be conducted face-to-face with the patients, their relatives and the ACP facilitators in the participants’ native language, audio-taped and integrally transcribed anonymising all personal details. The interview grid will be constructed following both an inductive and a deductive approach. The initial grid will be deducted from the research team’s experience in the topic and the literature. Five pilot interviews will be carried out to test the interview design. Whether the pilot interviews will be included in the dataset will be decided depending on the evaluation of their transcripts.

Video and audio data will serve for further analysis in subproject 3.

#### Data analysis

For data analysis, please see below at subproject 2b.

### Subproject 2b

To address the aims of subproject 2b, a qualitative study will be used based on semistructured interviews with patients and one of their relatives regarding the patient’s expression of their wish to die by assisted suicide. Participants will be recruited in the French, German and Italian-speaking parts of Switzerland.

#### Inclusion criteria for patients

- Expression of wish to die by assisted suicide.
- Being registered to a right to die association in Switzerland.
- 18 years or older.
- Capable of participating in an interview in French or Italian or German.

#### Inclusion criteria for relatives

- Being nominated and recruited by the patient.
- Having been informed by the patient of their intention to obtain assistance in suicide.
- 18 years or older.
- Capable of participating in an interview in French or Italian or German.

#### Exclusion criteria for patients and relatives

- Evidence of psychiatric symptoms or cognitive impairment that might significantly alter the decision-making capacity.

#### Procedure and sample size

Recruitment will take place through different sources: right to die associations and providers operating in the field of specialised palliative care. Snowball sampling will be also used. 10 patients and 10 relatives from the French-speaking part of Switzerland, 10 patients and 10 relatives from the German-speaking part of Switzerland, and 5 patients and 5 relatives from the Italian-speaking part of Switzerland will be recruited.

We intend to disseminate information about the study through two main channels:

- Specialised palliative care centres of the university hospitals in the French and German parts of Switzerland, and two palliative care centres of the Italian part of Switzerland. Recruitment will be non-systematic: health professionals within these services will inform the study investigator when a patient meets the criteria.

Right to die societies will inform their members actively pursuing assisted suicide decision about the study.

- Interviews will be conducted face-to-face with both the patients and their relatives. The interview grid will be constructed following the same procedure as described for subproject 2a.

#### Data analysis for subprojects 2a and 2b

The approach for data analysis is the same for subprojects 2a and 2b. To account for language and cultural differences, each coding step will be conducted in the regional language in which data were collected and data will then be shared through regular collaborative meetings. To ensure accuracy and quality in data generated, results will be translated into English and merged for a final transversal analysis.

The analysis will follow an inductive paradigm derived from thematic analysis.^44^ The interviews will be fragmented into significant text units, to which codes, or designations able to synthetically account for their content, will be assigned. The identified codes will be linked and grouped into larger categories to define more abstract concepts around which to organise the various arguments. 25% of the material will be double-coded independently by another researcher affiliated to the project, to allow for parallel coding. For subproject 2a, each data subset (ACP encounters and follow-up interviews) will be analysed individually as well as jointly. These operations will be made with the support of the specialised software for qualitative data analysis MAXQDA.

Our analysis will also account for the fact that the decisions involved in these subsets of data (ACP and assisted suicide) have important ethical implications. We will therefore undertake further conceptual analysis inspired by methods from analytical philosophy and the frameworks of the principles of biomedical ethics^45^ and of care ethics.^46^ Normative criteria will be identified to ethically evaluate altruistic acts at the end of life, based on the principles of biomedical ethics.^45^ Finally, we will present practical recommendations that promote the coexistence of patient autonomy and patient altruism.

### Subproject 3

#### Procedure and sample size

This subproject relies on natural data: audio and/or video-recorded naturally occurring interactions, taking place spontaneously, that is, not generated for the purpose of this study. Data will be collected in two settings in the French part of Switzerland.

1. ACP encounters

We will use data recorded for the subproject 2a.

2. Hospital palliative care units

We will record interactions taking place during 40 patient hospital admissions in three palliative centres in French Switzerland. This concerns activities taking place in the patient’s room, involving the patient, their visitors and palliative care professionals participating in the study. The term ‘activity’ will be used in the broad sense, comprising verbal and non-verbal exchanges and acts of care. Participants will be offered the option to decide, in advance, what type of activities they agree to be recorded in; consent will be reconfirmed prior to each recording. Participants will be offered the option of consenting to audio and video, or just to audio-recording.

#### Inclusion criteria for patients

- Hospitalised in one of three palliative care centres.
- Medically stable or when their state has improved.

#### Exclusion criteria for patients

- Imminence of death according to the referent physician.
- Impaired decision-making capacity.
- Presence of psychological/psychiatric problems due to which participating (being recorded) might harm the patient.

#### Inclusion criteria for healthcare professionals

- All health and allied health professionals affiliated to the palliative care unit.

#### Inclusion criteria for visitors and relatives

- ⁃Participation is open to all visitors of patients participating in the study.

#### Data analysis

Analysis will be guided by the conversation analysis (CA) approach. CA resides in a finely grained analysis of recorded data, focusing on how participants interact in order to accomplish ordinary as well as interactionally challenging tasks.^47^ While CA is an inductive approach, its use is regulated by a well-defined and stepwise process:

- The first-stage analysis is done as an ‘initial noticing’, in order to identify details of talk (‘phenomena’) that are interesting from a research point of view but also recurrent throughout the data.^48^
- Second, the researcher starts an exhaustive search throughout all the instances in which the phenomena occur and gathers them in datasets. Sequences of talk in which the phenomena are identified are transcribed according to a CA convention system designed for linguistic and multimodal transcriptions, which takes into account aspects of speech delivery and representation of activities parallel to talk (eye gaze, laughing, motions).^49–51^
- Third, the essential part of the analysis involves describing the phenomena in terms of sequential location (where it appears, why, what it generates) and content. The analysis will particularly draw on concepts of ‘affiliation’,^52^ ‘benefactors and beneficiaries’^53^ and empathic communication^54^ developed in CA and in relation to the field of palliative care.^55–57^

Data collected in the first setting (ACP conversations) will be monitored for how patients participate in the discussion of medical decisions, especially how patients may be ‘prosocial’ by orienting themselves towards others when talking, for example, about death-related topics, without being required to do so. This is in keeping with our definition of altruism.

Data collected in the second setting (hospital palliative care) will be monitored for when and how patients might interact (verbally or non-verbally) without being prompted, and about what. The focus will be on localising and analysing sequences of interaction in which palliative patients interact by their own volition. These instances will be investigated focusing on whether the patient’s involvement might be identified as being done for the benefit of the other person (eg, make a compliment, participate in an act of care or an exchange without being asked/required to).

### Patient and public involvement

No patients or members of the public were involved in the design of the study for any of the subprojects.

### Ethics and dissemination

The study project was approved by the Ethics Committees of the Canton of Vaud, Bern and Ticino (no: 2023-00088). Each project will be the subject of two scientific publications in peer-reviewed journals. Additional publications will be realised according to result triangulation between projects. The results will also be presented at international and national conferences. Finally, a symposium opened to professionals, patients and the public will be organised in Switzerland at the end of the project to present all the results.

## Supplementary Material

Reviewer comments

Author's manuscript
