# Supplementary material for: Interdisciplinary research approach based on a mixed-methods design to explore patient altruism at the end of life: a study protocol
Source: BMJ Open. 2024 May 10;14(5):e085632. doi: 10.1136/bmjopen-2024-085632 (PMC11097832; doi:10.1136/bmjopen-2024-085632)
Supplement: Author's manuscript [file bmjopen-2024-085632.draft_revisions.pdf]

BMJ Open

BMJ Open is committed to open peer review. As part of this commitment we make the peer review history of every article we publish publicly available.

When an article is published we post the peer reviewers' comments and the authors' responses online. We also post the versions of the paper that were used during peer review. These are the versions that the peer review comments apply to.

The versions of the paper that follow are the versions that were submitted during the peer review process. They are not the versions of record or the final published versions. They should not be cited or distributed as the published version of this manuscript.

BMJ Open is an open access journal and the full, final, typeset and author-corrected version of record of the manuscript is available on our site with no access controls, subscription charges or pay-per-view fees (http://bmjopen.bmj.com).

If you have any questions on BMJ Open's open peer review process please email info.bmjopen@bmj.com

BMJ Open

Understanding patient altruism at the end of life: An interdisciplinary research approach based on a mixed methods design

Journal:	*BMJ Open*
Manuscript ID	bmjopen-2024-085632
Article Type:	Protocol
Date Submitted by the Author:	21-Feb-2024

Complete List of Authors:	Bernard, Mathieu; Lausanne University Hospital, Palliative and Supportive Care Service<br>Gamondi, Claudia; Lausanne University Hospital, Palliative and Supportive Care Service<br>Sterie, Anca-Cristina; Lausanne University Hospital<br>Larkin, Philip J; Lausanne University Hospital<br>Jox, Ralf; Lausanne University Hospital, Palliative and Supportive Care Service<br>Borasio, Gian Domenico; Lausanne University Hospital, Palliative and Supportive Care Service

Keywords:	PALLIATIVE CARE, Adult palliative care < PALLIATIVE CARE, SOCIAL MEDICINE

SCHOLARONE™
Manuscripts

Understanding patient altruism at the end of life: An interdisciplinary research approach based on a mixed methods design

Mathieu Bernard[1], Claudia Gamondi[2], Ralf J. Jox[3], Phil Larkin[4], Anca-Cristina Sterie[2, 3], Gian Domenico Borasio[2]

[1] Palliative and Supportive Care Service, Chair of Palliative Psychology, Lausanne University Hospital and University of Lausanne, Switzerland

[2] Palliative and Supportive Care Service, Lausanne University Hospital and University of Lausanne, Switzerland

[3] Palliative and Supportive Care Service, Chair of Geriatric Palliative Care, Lausanne University Hospital and University of Lausanne, Switzerland

[4] Palliative and Supportive Care Service, Chair of Palliative Care Nursing, Lausanne University Hospital and University of Lausanne, Switzerland

Corresponding author:

Prof. Mathieu Bernard
Palliative and Supportive Care Service
Lausanne University Hospital
Avenue Pierre Decker 5
1011 Lausanne
Switzerland
Email: Mathieu.bernard@chuv.ch

Word count: 4667

Key words: palliative care, mixed methods, altruism, interdisciplinary

For peer review only - http://bmjopen.bmj.com/site/about/guidelines.xhtml

Abstract

Introduction:

In the end-of-life context, patients are often seen as somewhat passive recipients of care provided by health professionals and relatives, with little opportunity to be perceived as autonomous and active agents. Since studies show a very high prevalence of altruistic dispositions in palliative care patients, we aim strive to investigate the concept of patient altruism in a set of six interdisciplinary studies by considering three settings: (1) in the general palliative context, by studying to what extent patient altruism is associated with essential psychological outcomes of palliative care (subproject 1a), how altruism is understood by patients (subproject 1b), and how altruism expressed by patients is experienced by palliative care nurses (subproject 1c); (2) in two concrete decision-making contexts: advance care planning (subproject 2a) and assisted suicide (subproject 2b); and (3) through verbal and nonverbal patient communication in palliative care settings (subproject 3)

Methods and analysis

- Subproject 1a: a cross-sectional study using validated and standardized questionnaires
- Subprojects 1b and 1c: a constructivist grounded theory method aiming at developing a novel theory from semi-structured interviews in both patients and nurses.
- Subproject 2a: a thematic analysis based on (i) audio-recordings of advance care planning encounters and (ii) follow-up semi-directive interviews with patients and their relatives
- Subproject 2b: a qualitative study based on thematic analysis of interviews with patients actively pursuing assisted suicide and one of their relatives.
- Subproject 3: a conversation analysis based on audio and video-recorded interactions in two settings: (i) palliative inpatient unit and (ii) advance care planning discussions.

Ethics and dissemination

The study project was approved by the Ethics Committees of the Canton of Vaud, Bern and Ticino (n° 2023-00088).

Registration details

This work is supported by the Swiss National Science Foundation [grant number 10001G_207814 / 1].

Strengths and limitations

- We propose a set of multidisciplinary, complementary, and interlinked studies to explore altruism in palliative care in three different settings
- A complex concept such as patient altruism can only be addressed if several disciplines and methods are used
- Each subproject is conceived to explore a specific facet of patient altruism at the end of life and relies on a specific methodology of analysis corresponding to their research questions
- A joint phase of data and results triangulation between the subprojects will allow exchanges between teams on results and new interpretations of phenomena
- As a limitation, patients were not involved in the design of the study

For peer review only - http://bmjopen.bmj.com/site/about/guidelines.xhtml

INTRODUCTION

Palliative care challenges

In palliative care, the psychological domain is an essential component of patient care. Previous research in the field has mostly focused on improving the pharmacological and psychological treatments for the most frequent psychopathologies, i.e., anxiety, depression and adjustment disorders (1, 2). Less is known concerning the factors that foster psychological well-being, autonomy, and self-determination, thus improving quality of life. This approach, which has been termed "positive psychology" (3) parallels the resource-oriented, health-promoting approach known as salutogenesis. In recent years, palliative care patients have been considered as active participants in their own dying, but only concerning specific and well-defined aspects of the end of life, namely the so-called decisions concerning end-of-life (i.e. advance directives, withholding and withdrawing life-sustaining treatment, and requests for hastened death/assisted suicide where legally possible). In all other aspects concerning the last phase of life, patients are rather regarded as passive recipients of care, with only little opportunity to be perceived as "agents" who act, give, and contribute something by and of themselves. One possible way of fostering patient autonomy and well-being at the end of life is to consider the patient in line with the concept of "patient empowerment". While this paradigm is trending in many medical disciplines (4), it has had little impact in the field of palliative care so far, with the exception of those situations where decisions need to be made. It is precisely for this reason that we place the concept of patient altruism, as a form of patient agency, at the center of this project.

Nowadays, the concept of altruism is used in various disciplines such as psychology, philosophy, economics, sociology, and evolutionary biology (5, 6). Numerous authors tend to apply this concept to any prosocial behavior or action carried out on a voluntary basis and aiming to benefit the society at large, specific individuals or a group of individuals (5, 7-9). While altruistic behavior is primarily explained as an individual constitutive characteristic (10-13), this behavior has to be understood by considering its sources of motivation (benevolence (11), empathy (14), reward (15), norms (16), and anger (5, 17)). Social norms and interaction rules (social responsibility, group gain, reciprocity, negotiated rules) have also been identified as determinants of altruistic behaviors (18). To adequately address this multi-level concept (personality, motivations, social norms) an interdisciplinary approach appears necessary, which is also underscored by the different disciplines involved in this project (humanities, social sciences, medical sciences and nursing).

Although palliative care addresses all people suffering from a life-limiting illness, most palliative care patients who benefit from it are close to end of their life and are older of age. It is well established that older people report higher altruism levels than younger people (19). This is in agreement with life span developmental theories that have shown a change in motivation orientation during the last phase of existence, which promotes a sense of realization and meaning in life (20) . While meaning in life tends to increase with age (21, 22), the sources of meaning change: Sparrow et al. showed that, with increasing age, intrinsic values (such as authenticity, intimacy, spirituality but also altruism), are prioritized over extrinsic goals, such as achievement, competence, and power (23). Other authors mention a shift towards meaningful social goals focusing on others, especially close ones, after the recognition that time is becoming more limited as age increases (24). Vollhardt suggests that altruism may also be a consequence of suffering after adverse life events, such as a life-threatening illness (25, 26). In

For peer review only - http://bmjopen.bmj.com/site/about/guidelines.xhtml

line with these findings, Fegg et al. showed that palliative care patients consistently reported higher self-transcendent and altruistic values as compared to healthy adults (27). In addition, palliative care patients cite the social dimension as a source of meaning in life more often than the general population (28).

Since the fragility of patients at the end of life often reduces their capacity to undertake concrete altruistic actions by themselves, we need to broaden our understanding of altruism by also taking into account the attitudes and values at the origin of altruistic behaviors. In the theoretical model of basic human values developed by Schwartz, the values of "benevolence" and "universalism" ("self-transcendence") refer to concern for others' welfare and represent an internal motivation promoting social relations (29, 30). Since there is currently no clear definition or conceptual framework of altruism that has been applied to palliative care patients, we propose to define altruism in this context as: "a personal and intentional interest in improving the wellbeing and welfare of others – at an individual, group or societal level". By emphasizing the notion of "interest", we aim to include the different levels of manifestations of altruism that might be relevant to palliative care, including the (advance) decisions made by patients and the interactions upon which these decisions are based.

We therefore propose a set of multidisciplinary, complementary, and interlinked studies to explore altruism in palliative care in three different settings:

- The first level refers to patient altruism in the general context of palliative care:
  - We first aim to study to what extent altruism, considered in terms of prosocial behavior and self-transcendent value, is associated with important outcomes and indicators of palliative care (subproject 1a).
  - We then aim to explore (i) how altruism is defined and understood by the palliative care patients themselves (subproject 1b) and (ii) how altruism from patients is experienced by palliative care nurses, the professionals who are closest to patients in clinical practice, and how the expression of altruism by patients and families towards nurses influences professional meaning and fulfillment (subproject 1c).
- The second level considers how altruism might be expressed and realized through specific decisions between patients and their social environment by considering two specific decision-making contexts of high importance for palliative care: advance care planning (ACP, subproject 2a) and assisted suicide (subproject 2b). Both represent profound existential expressions of autonomy, which are closely related to the patients' values and attitudes.
- The third level concerns how altruism might be expressed in social interactions (both verbal or non-verbal), and how the interaction itself might have an altruistic dimension. We consider moments in which patients are involved in interaction by their own volition, and in which such interaction might bring a potential benefit to the other (subproject 3).

This project is anchored in an interdisciplinary effort bringing together the expertise of several researchers in palliative healthcare. They are therefore representative of the interdisciplinary nature of palliative care, as defined by the WHO (31). Mathieu Bernard, head of sub-projects 1a and 1b, is a psychologist; Phil Larkin, head of sub-project 1c, is a nurse; Ralf Jox, head of sub-project 2a, and Claudia Gamondi, head of sub-project 2b, are physicians, as Gian Domenico

For peer review only - http://bmjopen.bmj.com/site/about/guidelines.xhtml

Borasio, project coordinator. Finally, Anca-Cristina Sterie, head of sub-project 3, is a sociologist specialized in interactions in the medical setting.

The repercussions of this project could be manifold: first, it would allow designing and testing an altruism-based intervention for palliative care patients that could represent an important new step in the development of efficient, resource-oriented palliative care. Secondly, such interventions would have the potential to restore dignity and autonomy for patients in the last phase of life by allowing them, if they so wish, to assume a more active role. Thirdly, the expression of patient altruism towards family members and health care professionals could also profoundly affect the latter two: it could diminish their distress and ease their grieving, improve relationships and may be a role model for themselves to become more altruistic.

METHODS

Subproject 1a

For peer review only

A cross-sectional study using validated and standardized questionnaires will be conducted in both the French- and German-speaking parts of Switzerland (two University Hospitals and five palliative care centers), and two palliative care centers in the Italian-speaking part of Switzerland.

Inclusion criteria:

- 18 years or older
- treated by one of the palliative care teams
- <6 months life expectancy according to the treating physician
- medically stable state or when their state has improved

Exclusions criteria:

- evidence of psychiatric and cognitive symptoms that might significantly alter the decision-making capacity
- insufficient knowledge of the local language

Questionnaires and procedure

For the purpose of the study, we will use:

- The Prosocialness Scale for Adults (PSA) (32)
- The benevolence (four items) and universalism (six items) subscales of the 40-item Portrait Values Questionnaire (PVQ) (30)
- Quality of life will be measured with the McGill Quality of Life scale Revised version (MQOL-R) (33)
- Psychological distress will be measured with the Hospital Anxiety and Depression Scale (HADS) (34)
- Patient's feeling of being a burden to their caregivers will be assessed with the Self Perceived Burden Scale (SPBS) (35)
- Meaning in life will be assessed with the Meaning in Life Questionnaire (MLQ) (36)
- The will to live intensity will be measured with a single-item numerical rating scale (37)

These questionnaires were chosen because they measure dimensions that have been identified as psychological determinants of quality of life in palliative care. All new patients admitted in the palliative care centres participating in the study and who fulfil the inclusion and exclusion

For peer review only - http://bmjopen.bmj.com/site/about/guidelines.xhtml

criteria as assessed by the referring physician, will be asked by a research assistant to participate in the study as soon as an appropriate acute symptom control has been reached. Written informed consent will be obtained.

Validated translations or cross-cultural adaptations of the questionnaires will be used in face-to-face interviews. In addition, sociodemographic variables and medical data (principal diagnosis, co morbidities, performance status) will be obtained. Finally, questions using numerical rating scales (0-10) will assess the level of stress and the potential for personal development induced by the questionnaires. Such numerical rating scales will also be used to assess (i) to what extent altruism is an important concept in the end-of-life context, and (ii) to what extent patients feel frustration not to be able to express altruism towards others.

Data analysis strategy

Rates will be reported for the recruitment information. Descriptive statistics will be calculated for socio-demographic variables and all outcomes. Missing outcome data will be treated as mentioned in the scoring manuals. Distribution of each outcome measures will be analysed using normality tests and other indicators of distribution (box-plots, skewness and kurtosis estimations). Associations between outcome variables will be assessed using Pearson or Spearman correlations according to distributions. Univariate and multiple linear regressions will be performed in order to determine whether altruism can be considered as a significant explanatory variable (in addition to psychological distress, desire to live, feeling of being a burden and meaning in life) for quality of life. We will use an alpha threshold <.05 as a criterion for rejecting the null hypothesis. All models will be controlled for socio-demographic characteristics.

Sample size

Multiple linear regressions represent the crucial elements for assessing the number of participants to be included to ensure sufficient power. According to Howell (38), we aim to recruit 15 patients per explanatory variable, i.e. 120 patients with complete data in this study (50 in the French- and German-speaking parts and 20 in the Italian-speaking part, according to population representativeness). Estimating an exclusion rate of 60% and a refusal rate of 50% based on previous studies in the same context (39-41), 600 patients in total will need to be screened for study eligibility (250 in the French- and German-speaking parts and 100 in the Italian-speaking part).

Subproject 1b

Procedure and sample size

A subsample of participants from project 1a will be invited to participate in subproject 1b. Agreement to participate will be sought at the end of project 1a data collection. We estimate a maximum sample of 45 participants (approximately 15 per linguistic region), depending on data saturation (REF) and the need to explore items in greater or lesser depth. Therefore, the final sample may not be determined until the end of the study. We will aim to maximise the sample variation by ensuring diversity across gender, age, setting of care, illness type (e.g. cancer vs. non-cancer), and also by considering the score on the two questionnaires assessing altruism.

A constructivist grounded theory method will be adopted for project 1b and 1c (42). In this qualitative method the researcher inductively analyses individual and collective actions as well

For peer review only - http://bmjopen.bmj.com/site/about/guidelines.xhtml

For peer review only

as social and psychological processes, approaching the data with no preconceived ideas or hypotheses, accepting that there may be multiple perspectives and acknowledging the importance of their own place in constructing those perspectives with participants. Data collection and analysis are undertaken in parallel and decisions on sampling are revised as the project develops, a process referred to as 'theoretical sampling'.

An initial interview guide will be developed based on topics derived from data collected in sub-project 1a and from research questions of subproject 1b. Semi-structured interviews will be conducted by a researcher trained in qualitative methodology and grounded theory data analysis, supported by qualitative research experts. The semi-structured approach enables the interviewer to vary the wording and sequence of the questions and pursue leads provided by the participants, while being respectful of the burden of interviewing a frail and potentially vulnerable population.

The interview guide will be developed by the research team, comprising native speakers in French, German, Italian and English. The team will meet after the first five interviews in each region to discuss emerging themes and agree if new directions in terms of revised questions are needed. Theoretical saturation will be determined first in each region and later agreed through consensus with the wider research team.

Data analysis

All interviews will be digitally recorded and transcribed in the language of origin. To account for language and cultural differences, interpretation and analysis of the data will be carried out by research collaborators in each region, trained in qualitative methodology and supported by the local research partners. Data will be entered into MAXQDA software to assist analysis.

In accordance with constructivist grounded theory methods, a broad pool of codes will be obtained during an open (initial) coding using interview data, notes and memos. In a second phase of axial (focused) coding, all individual codes will be sorted and resorted as concepts and common themes are developed (42). In the final step, (theoretical coding) overarching themes will be grouped into more refined categories and central concepts, again referring to wider data sources (notes, memos, etc.). Each coding step will be conducted in the individual language for the region and data shared through regular collaborative meetings to ensure accuracy and quality. An iterative discussion process will be engaged throughout the study, in order to reach a substantial agreement between researchers in terms of major themes and any cultural or regional difference identified. The final themes will be translated into English as the common language for the presentation of data and subjected to a second level inductive analysis and comparison with the host language for accuracy in terms of conceptual equivalence. The emerging theory will be co-constructed by the research team.

Subproject 1c

Procedure and sample size

We first begin with a conceptual analysis of altruism to determine its antecedents, attributes and relationships with palliative care nursing. Conceptual analyses are of particular benefit where a concept requires refinement within a specific discipline or context (43). This will also inform the development of the interview guide and the outcome can be refined during interviews with the nursing participants.

For peer review only - http://bmjopen.bmj.com/site/about/guidelines.xhtml

Following the constructivist grounded theory principles outlined in subproject 1b, 30 semi-structured in-depth interviews with nurses working in palliative care nursing practice will be undertaken. Participants will be accessed through the same University Hospitals and palliative care centres collaborating for subprojects 1a and 1b (French, German and Italian). Interviews will provide concrete examples of the nurses' experience of altruism in practice and address the meaning and impact of altruism for the palliative care nurse practitioner.

Inclusion criteria for nurses:

- Working at specialist or generalist level within an agreed palliative care setting
- Having worked in a full-time or part-time capacity for at least 6 months to be able to reflect and discuss their professional experience and offer concrete examples from practice.
- Willing to be interviewed and recorded
- Able to converse in either French, German, Italian or English

Since the division between specialist and generalist palliative care nursing roles is ill-defined, interviews with a broader nursing sample will help to understand the broader facilitators and barriers to altruism and to seek ulterior expressions and meanings of altruism within a wider professional nursing cohort. Ten interviews per linguistic region are planned.

Data analysis

The process of data analysis described in subproject 1b will also be applied to subproject 1c for symmetry and completeness of data across subprojects 1b and 1c. It will then afford opportunity to consider both data sets for similarities and disparities in the understanding and expression of altruism.

Subproject 2a

In order to address the aims of subproject 2a, a qualitative design will be used with three types of data; (i) audio or video-recorded routine ACP discussions between a facilitator, a patient and their relative(s) if applicable; (ii) semi-structured follow-up interviews of the persons having participated in these discussions.

Inclusion criteria for patients

- 18 years or older
- receiving general or specialized palliative care

Inclusion criteria for relatives

- being nominated and recruited by the patient
- 18 years or older
- capable to participate to an interview in French or German

Inclusion criteria for ACP facilitators

- acting as facilitator of ACP discussions with the patient (and his or her relatives)
- being trained as ACP facilitator

Exclusion criteria for patients and relatives

- evidence of psychiatric or cognitive symptoms significantly altering the decision-making capacity

For peer review only - http://bmjopen.bmj.com/site/about/guidelines.xhtml

Procedure and sample size

We will follow a sampling strategy combining cluster sampling and convenience sampling. The cluster sampling will be oriented towards variation regarding gender, age groups and health status of the patient participants (oncological, neurological and cardiorespiratory illnesses). The convenience part of the sampling relates to the fact that we will recruit in structured and professionally facilitated ACP activities in the University Hospitals of both the French and German parts of Switzerland, and in two palliative care centers of the Italian part of Switzerland. It is planned to conduct 9-12 qualitative interviews with ACP facilitators (3-4 per language region), and to register 24 audio-recorded routine ACP discussions (8 in each language area), as well as 24 follow-up interviews with patients and 24 follow-up interviews with relatives having participated in the ACP discussions.

Data collection

The ACP discussions will be audio or video recorded. Audio-recordings will be transcribed verbatim. The transcripts will then be coded by the investigator and analyzed by the researcher.

The semi-structured interviews will be conducted face-to-face with the patients, their relatives, and the ACP facilitators in the participants' native language, audio-taped and integrally transcribed anonymizing all personal details. The interview grid will be constructed following both an inductive and a deductive approach. The initial grid will be deducted from the research team's experience in the topic and the literature. Five pilot interviews will be carried out to test the interview design. Whether the pilot interviews will be included in the data set will be decided depending on the evaluation of their transcripts.

Video and audio data will serve for further analysis in subproject 3.

Data analysis

For data analysis, please see below at project 2b

Subproject 2b

To address the aims of subproject 2b, a qualitative study will be used based on semi-structured interviews with patients and one of their relatives regarding the patient's expression of their wish to die by assisted suicide. Participants will be recruited in the French, German, and Italian-speaking parts of Switzerland.

Inclusion criteria for patients

- expression of wish to die by assisted suicide
- being registered to a right-to-die association in Switzerland
- 18 years or older
- capable of participating in an interview in French or Italian or German

Inclusion criteria for relatives

- being nominated and recruited by the patient
- having been informed by the patient of their intention to obtain assistance in suicide
- 18 years or older
- capable of participating in an interview in French or

For peer review only - http://bmjopen.bmj.com/site/about/guidelines.xhtml

Exclusion criteria for patients and relatives

- evidence of psychiatric symptoms or cognitive impairment that might significantly alter the decision-making capacity.

Procedure and sample size

Recruitment will take place through different sources; right to die associations and providers operating in the field of specialised palliative care. Snowball sampling will be also used. Ten patients and 10 relatives from the French-speaking part of Switzerland, 10 patients and 10 relatives from the German-speaking part of Switzerland, and 5 patients and 5 relatives from the Italian-speaking part of Switzerland will be recruited.

We intend to disseminate information about the study through two main channels:

- Specialized palliative care centers of the University Hospitals in the French and German parts of Switzerland, and two palliative care centers of the Italian part of Switzerland. Recruitment will be non-systematic: health professionals within these services will inform the study investigator when a patient meets the criteria

Right-to-die societies that will inform their members actively pursuing assisted suicide decision about the study

- Interviews will be conducted face-to-face with both the patients and their relatives. The interview grid will be constructed following the same procedure as described for subproject 2a.

Data analysis for sub-projects 2a and 2b

The approach for data analysis is the same for sub-projects 2a and 2b. To account for language and cultural differences, each coding step will be conducted in the regional language in which data was collected and data will then be shared through regular collaborative meetings. To ensure accuracy and quality in data generated, results will be translated into English and merged for a final transversal analysis.

The analysis will follow an inductive paradigm derived from thematic analysis (44). The interviews will be fragmented into significant text units, to which codes, or designations able to synthetically account for their content, will be assigned. The identified codes will be linked and grouped into larger categories to define more abstract concepts around which to organize the various arguments. 25% of the material will be double coded independently by another researcher affiliated to the project, to allow for parallel coding. For subproject 2a, each data subset (ACP encounters and follow-up interviews) will be analyzed individually as well as jointly. These operations will be made with the support of the specialized software for qualitative data analysis MAXQDA.

Our analysis will also account for the fact that the decisions involved in these subsets of data (ACP and assisted suicide) have important ethical implications. We will therefore undertake further conceptual analysis inspired by methods from analytical philosophy and the frameworks of the principles of biomedical ethics (45) and of care ethics (46). Normative criteria will be identified to ethically evaluate altruistic acts at the end of life, based on the principles of biomedical ethics (45). Finally, we will present practical recommendations that promote the co-existence of patient autonomy and patient altruism.

For peer review only - http://bmjopen.bmj.com/site/about/guidelines.xhtml

Subproject 3

Procedure and sample size

This subproject relies on natural data: audio and/or video recorded naturally occurring interactions, taking place spontaneously, i.e., not generated for the purpose of this study. Data will be collected in two settings in the French part of Switzerland

1. Advance Care Planning encounters

We will use data recorded for the sub-project 2a.

2. Hospital palliative care units

We will record interactions taking place during 40 patient hospitalizations in three palliative centres in French Switzerland. This concerns activities taking place in the patient's room, involving the patient, their visitors, and palliative care professionals participating to the study. The term 'activity' will be used in the broad sense, comprising verbal and non-verbal exchanges and acts of care. Participants will be offered the option to decide, in advance, what type of activities they agree to be recorded in; consent will be reconfirmed prior to each recording. Participants will be offered the option of consenting to audio and video, or just to audio recording.

Inclusion criteria for patients

- hospitalized in one of three palliative care centres
- medically stable or when their state has improved

Exclusion criteria for patients

- imminence of death according to the referent physician
- impaired decision-making capacity
- presence of psychological/psychiatric problems due to which participating (being recorded) might harm the patient

Inclusion criteria for health care professionals

- All health and allied health professionals affiliated to the palliative care unit

Inclusion criteria for visitors and relatives

- Participation is open to all visitors of patients participating to the study

Data analysis

Analysis will be guided by the conversation analysis (CA) approach. CA resides in a finely-grained analysis of recorded data, focusing on how participants interact in order to accomplish ordinary as well as interactionally challenging tasks (47). While CA is an inductive approach, its use is regulated by a well-defined and stepwise process:

- The first-stage analysis is done as an "initial noticing", in order to identify details of talk ("phenomena") that are interesting from a research point of view but also recurrent throughout the data (48).

For peer review only - http://bmjopen.bmj.com/site/about/guidelines.xhtml

- Secondly, the researcher starts an exhaustive search throughout all the instances in which the phenomena occur and gathers them in data sets. Sequences of talk in which the phenomena are identified are transcribed according to a CA convention system designed for linguistic and multi-modal transcriptions, which takes into account aspects of speech delivery and representation of activities parallel to talk (eye gaze, laughing, motions) (49-51).
- Thirdly, the essential part of the analysis involves describing the phenomena in terms of sequential location (where it appears, why, what it generates) and content. The analysis will particularly draw on concepts of 'affiliation' (52), 'benefactors and beneficiaries' (53) and empathic communication (54) developed in CA and in relation to the field of palliative care (55-57).

Data collected in the first setting (ACP conversations) will be monitored for how patients participate to the discussion of medical decisions, especially how patients may be "pro-social" by orienting themselves towards others when talking e.g. about death-related topics, without being required to do so. This is in keeping with our definition of altruism.

Data collected in the second setting (hospital palliative care) will be monitored for when and how patients might interact (verbally or non-verbally) without being prompted, and about what. The focus will be on localizing and analyzing sequences of interaction in which palliative patients interact by their own volition. These instances will be investigated focusing on whether the patient's involvement might be identified as being done for the benefit of the other person (e.g. make a compliment, participate to an act of care or an exchange without being asked/required to).

Conclusion

The idea behind our joint venture is that a complex concept such as patient altruism at the end of life can only be addressed using several complementary disciplines and methods. While each of the six subprojects is an autonomous entity, we also consider them as interconnected since they are designed to form a coherent ensemble that crosscuts through several settings, linguistic regions, variables and phenomena related to patient altruism. Consequently, a joint phase of data and result triangulations for all subprojects is scheduled, which will allow exchanges between teams on results and new interpretations of observed phenomena. This project aims at opening a window onto a previously untapped resource for the improvement of the quality of life of palliative care patients by opening a space for them to express their own altruism towards the people they care for. Shifting the focus on patients' altruism could also change the way patients are generally seen in health care and research, and lead to a more resource-oriented, patient-centered health care approach.

ETHICS AND DISSEMINATION

The study project was approved by the Ethics Committees of the Canton of Vaud, Bern and Ticino (n°2023-00088). Each project will be the subject of two scientific publications in peer-reviewed journals. Additional publications will be realized according to result triangulations between projects. The results will also be presented at international and national conferences. Finally, a special symposium will be organized in Switzerland at the end of the project to present all the results.

For peer review only - http://bmjopen.bmj.com/site/about/guidelines.xhtml

REFERENCES

1. Mitchell AJ, Chan M, Bhatti H, Halton M, Grassi L, Johansen C, et al. Prevalence of depression, anxiety, and adjustment disorder in oncological, haematological, and palliative-care settings: a meta-analysis of 94 interview-based studies. The Lancet Oncology. 2011;12(2):160-74.
2. Parpa E, Tsilika E, Gennimata V, Mystakidou K. Elderly cancer patients' psychopathology: A systematic review Aging and mental health. Archives of Gerontology and Geriatrics. 2015;60(1):9-15.
3. Seligman, Csikszentmihalyi. Positive psychology. An introduction. Am Psychol. 2000;55(1):5-14.
4. Pekonen A, Eloranta S, Stolt M, Virolainen P, Leino-Kilpi H. Measuring patient empowerment - A systematic review. Patient Educ Couns. 2020;103(4):777-87.
5. Fehr E, Gächter S. Altruistic punishment in humans. Nature. 2002;415(6868):137-40.
6. Sonne JWH, Gash DM. Psychopathy to Altruism: Neurobiology of the Selfish-Selfless Spectrum. Frontiers in psychology. 2018;9:575.
7. FitzPatrick WJ. Human altruism, evolution and moral philosophy. Royal Society open science. 2017;4(8):170441.
8. Warneken F, Tomasello M. The roots of human altruism. British journal of psychology (London, England : 1953). 2009;100(Pt 3):455-71.
9. West SA, Gardner A, Griffin AS. Altruism. Current biology : CB. 2006;16(13):R482-3.
10. DeYoung CG, Quilty LC, Peterson JB. Between facets and domains: 10 aspects of the Big Five. Journal of personality and social psychology. 2007;93(5):880-96.
11. Hubbard J, Harbaugh WT, Srivastava S, Degras D, Mayr U. A general benevolence dimension that links neural, psychological, economic, and life-span data on altruistic tendencies. Journal of experimental psychology General. 2016;145(10):1351-8.
12. McCrae RR, John OP. An introduction to the five-factor model and its applications. Journal of personality. 1992;60(2):175-215.
13. Szuster A. Crucial Dimensions of Human Altruism. Affective vs. Conceptual Factors Leading to Helping or Reinforcing Others. Frontiers in psychology. 2016;7:519.
14. Batson CD, Batson JG, Slingsby JK, Harrell KL, Peekna HM, Todd RM. Empathic joy and the empathy-altruism hypothesis. Journal of personality and social psychology. 1991;61(3):413-26.
15. Carlo G, Hausmann A, Christiansen S, Randall BA. Sociocognitive and behavioral correlates of a measure of prosocial tendencies for adolescents. The Journal of Early Adolescence. 2003;23(1):107-34.
16. Chen H, Zeng Z, Ma J. The source of punishment matters: Third-party punishment restrains observers from selfish behaviors better than does second-party punishment by shaping norm perceptions. PloS one. 2020;15(3):e0229510.
17. Mussweiler T, Ockenfels A. Similarity increases altruistic punishment in humans. Proceedings of the National Academy of Sciences of the United States of America. 2013;110(48):19318-23.
18. Cropanzano R, Mitchell MS. Social exchange theory: An interdisciplinary review. [References]. Journal of Management. 2005;31(6):874-900.
19. Sparrow EP, Swirsky LT, Kudus F, Spaniol J. Aging and altruism: A meta-analysis. Psychology and aging. 2021;36(1):49-56.
20. Dunkel CS, Harbke C. A review of measures of Erikson's stages of psychosocial development: Evidence for a general factor. Journal of Adult Development. 2017;24(1):58-76.
21. Reker GT, Wong PTR. Personal meaning in life and psychosocial adaptation in the later years. [References]. In: Wong PTP, editor. The human quest for meaning: Theories, research, and applications. 2nd ed. New York, NY, US: Routledge/Taylor & Francis Group; US.; 2012. p. 433-56.
22. Reker GT, Woo LC. Personal meaning orientations and psychosocial adaptation in older adults. [References]: SAGE Open. Vol.1(1), 2011, pp. 1-10.; 2011.
23. Sparrow EP, Spaniol J. Aging and altruism in intertemporal choice. Psychology and aging. 2018;33(2):315-24.

For peer review only - http://bmjopen.bmj.com/site/about/guidelines.xhtml

24. Carstensen LL, Fung HH, Charles ST. Socioemotional selectivity theory and the regulation of emotion in the second half of life. [References]. Motivation and Emotion. 2003;27(2):103-23.
25. Staub E, Vollhardt J. Altruism born of suffering: the roots of caring and helping after victimization and other trauma. The American journal of orthopsychiatry. 2008;78(3):267-80.
26. Vollhardt JR. Altruism born of suffering and prosocial behavior following adverse life events: A review and conceptualization. [References]. Social Justice Research. 2009;22(1):53-97.
27. Fegg MJ, Wasner M, Neudert C, Borasio GD. Personal values and individual quality of life in palliative care patients. J Pain Symptom Manage. 2005;30(2):154-9.
28. Bernard M, Berchtold A, Strasser F, Gamondi C, Borasio GD. Meaning in life and quality of life: palliative care patients versus the general population. BMJ Support Palliat Care. 2020.
29. Sagiv L, Roccas S, Cieciuch J, Schwartz SH. Personal values in human life. Nature human behaviour. 2017;1(9):630-9.
30. Schwartz SH, Cieciuch J. Measuring the Refined Theory of Individual Values in 49 Cultural Groups: Psychometrics of the Revised Portrait Value Questionnaire. Assessment. 2021:1073191121998760.
31. World Health Organisation. Global atlas of palliative care at the end of life 2014 [Available from: http://www.who.int/cancer/palliative/definition/en/.
32. Caprara GV, Steca P, Zelli A, Capanna C. A new scale for measuring adults' prosocialness. European Journal of Psychological Assessment. 2005;21(2):77-89.
33. Cohen SR, Sawatzky R, Russell LB, Shahidi J, Heyland DK, Gadermann AM. Measuring the quality of life of people at the end of life: The McGill Quality of Life Questionnaire-Revised. Palliat Med. 2017;31(2):120-9.
34. Zigmond AS, Snaith RP. The hospital anxiety and depression scale. Acta psychiatrica Scandinavica. 1983;67(6):361-70.
35. Cousineau N, McDowell I, Hotz S, Hébert P. Measuring chronic patients' feelings of being a burden to their caregivers: development and preliminary validation of a scale. Medical care. 2003;41(1):110-8.
36. Naghiyaee M, Bahmani B, Asgari A. The Psychometric Properties of the Meaning in Life Questionnaire (MLQ) in Patients with Life-Threatening Illnesses. TheScientificWorldJournal. 2020;2020:8361602.
37. Bornet MA, Bernard M, Jaques C, Rubli Truchard E, Borasio GD, Jox RJ. Assessing the Will to Live: A Scoping Review. J Pain Symptom Manage. 2021;61(4):845-57.e18.
38. Howell CA. Statistical methods for psychology. Seventh edition ed. Belmont, CA, USA: Cengage Wadsworth; 2017.
39. Althaus B, Borasio GD, Bernard M. Gratitude at the End of Life: A Promising Lead for Palliative Care. J Palliat Med. 2018;21(11):1566-72.
40. Bernard M, Strasser F, Gamondi C, Braunschweig G, Forster M, Kaspers-Elekes K, et al. Relationship Between Spirituality, Meaning in Life, Psychological Distress, Wish for Hastened Death, and Their Influence on Quality of Life in Palliative Care Patients. J Pain Symptom Manage. 2017;54(4):514-22.
41. Brenner KO, Rosenberg LB, Cramer MA, Jacobsen JC, Applebaum AJ, Block SD, et al. Exploring the Psychological Aspects of Palliative Care: Lessons Learned from an Interdisciplinary Seminar of Experts. J Palliat Med. 2021;24(9):1274-9.
42. Charmaz K. Constructing grounded theory. 2nd ed. Thousand Oaks, CA, US: Sage publications; 2014.
43. Sharifi N, Adib-Hajbaghery M, Najafi M. Cultural competence in nursing: A concept analysis. Int J Nurs Stud. 2019;99:103386.
44. Braun V, Clarke V. What can "thematic analysis" offer health and wellbeing researchers? Int J Qual Stud Health Well-being. 2014;9:26152.
45. Beauchamp T, Childress J. Principles of Biomedical Ethics: Marking Its Fortieth Anniversary. Am J Bioeth. 2019;19(11):9-12.
46. Gilligan C. In A Different Voice. Cambridge: Harvard University Press; 1982.

For peer review only - http://bmjopen.bmj.com/site/about/guidelines.xhtml

47. Schegloff EA. Sequence organization in interaction: A primer in conversation analysis I: Cambridge university press; 2007.
48. Schegloff EA. Confirming allusions: Toward an empirical account of action. American journal of sociology. 1996;102(1):161-216.
49. Jefferson G. Glossary of transcript symbols with an introduction. In: Lerner GH, editor. Conversation Analysis: Studies from the first generation: John Benjamins; 2004. p. 13-34.
50. Parry R. Video-based conversation analysis. Sage handbook of qualitative methods in health research. London: Sage; 2010. p. 373-96.
51. Rossano F. Gaze in Conversation. In: Sidnell J, Stivers T, editors. The handbook of conversation analysis: Blackwell Publishing; 2013. p. 308-29.
52. Steensig J. Conversation analysis and affiliation and alignment. The encyclopedia of applied linguistics: Wiley; 2012. p. 1-6.
53. Clayman SE, Heritage J. Benefactors and beneficiaries: Benefactive Status and Stance in the Management of Offers and Requests. In: Couper-Kuhlen E, Drew P, editors. Requesting in social interaction. Amsterdam: John Benjamins; 2014. p. 55-86.
54. Heritage J, Maynard DW. Communication in medical care: Interaction between primary care physicians and patients: Cambridge University Press; 2006.
55. Gramling D, Gramling R. Palliative care conversations: clinical and applied linguistic perspectives: Walter de Gruyter GmbH & Co KG; 2019.
56. Jenkins L, Parry R, Pino MJAL. Providing opportunities for patients to say more about their pain without overtly asking: a conversation analysis of doctors repeating patient answers in palliative care pain assessment. Applied Linguistics. 2021:1-24.
57. Land V, Parry R, Pino M, Jenkins L, Feathers L, Faull C. Addressing possible problems with patients' expectations, plans and decisions for the future: One strategy used by experienced clinicians in advance care planning conversations. Patient Education and Counseling. 2019;102(4):670-9.

AUTHORS' CONTRIBTIONS

MB and GDB designed the study protocol. MB developed the study design and method of subproject 1a; MB and PL developed the study design and method OF subproject 1b; PL developed the study design and method of subproject 1c; RJ developed the study design and method of subproject 2a; CG developed the study design and method of subproject 2b; AS developed the study design and method of subproject 3.

FUNDING STATEMENT

This work is supported by the Swiss National Science Foundation [grant number 10001G_207814 / 1]. The study is funded for a three years period from April 2023 to March 2026.

For peer review only

BMJ Open

An interdisciplinary research approach based on a mixed methods design to explore patient altruism at the end of life: a study protocol

Journal:	*BMJ Open*
Manuscript ID	bmjopen-2024-085632.R1
Article Type:	Protocol
Date Submitted by the Author:	09-Apr-2024
Complete List of Authors:	Bernard, Mathieu; Lausanne University Hospital, Palliative and Supportive Care Service<br>Gamondi, Claudia; Lausanne University Hospital, Palliative and Supportive Care Service<br>Sterie, Anca-Cristina; Lausanne University Hospital<br>Larkin, Philip J; Lausanne University Hospital<br>Jox, Ralf; Lausanne University Hospital, Palliative and Supportive Care Service<br>Borasio, Gian Domenico; Lausanne University Hospital, Palliative and Supportive Care Service
**Primary Subject Heading**:	Palliative care
Secondary Subject Heading:	Qualitative research
Keywords:	PALLIATIVE CARE, Adult palliative care < PALLIATIVE CARE, SOCIAL MEDICINE

SCHOLARONE™
Manuscripts

An interdisciplinary research approach based on a mixed methods design to explore patient altruism at the end of life: a study protocol

Mathieu Bernard[1], Claudia Gamondi[2], Ralf J. Jox[3], Phil Larkin[4], Anca-Cristina Sterie[2, 3], Gian Domenico Borasio[2]

[1] Palliative and Supportive Care Service, Chair of Palliative Psychology, Lausanne University Hospital and University of Lausanne, Switzerland

[2] Palliative and Supportive Care Service, Lausanne University Hospital and University of Lausanne, Switzerland

[3] Palliative and Supportive Care Service, Chair of Geriatric Palliative Care, Lausanne University Hospital and University of Lausanne, Switzerland

[4] Palliative and Supportive Care Service, Chair of Palliative Care Nursing, Lausanne University Hospital and University of Lausanne, Switzerland

Corresponding author:

Prof. Mathieu Bernard
Palliative and Supportive Care Service
Lausanne University Hospital
Avenue Pierre Decker 5
1011 Lausanne
Switzerland
Email: Mathieu.bernard@chuv.ch

Word count: 4527

Key words: palliative care, mixed methods, altruism, interdisciplinary

For peer review only - http://bmjopen.bmj.com/site/about/guidelines.xhtml

Abstract

Introduction:

In the end-of-life context, patients are often seen as somewhat passive recipients of care provided by health professionals and relatives, with little opportunity to be perceived as autonomous and active agents. Since studies show a very high prevalence of altruistic dispositions in palliative care patients, we aim strive to investigate the concept of patient altruism in a set of six interdisciplinary studies by considering three settings: (1) in the general palliative context, by studying to what extent patient altruism is associated with essential psychological outcomes of palliative care (subproject 1a), how altruism is understood by patients (subproject 1b), and how altruism expressed by patients is experienced by palliative care nurses (subproject 1c); (2) in two concrete decision-making contexts: advance care planning (subproject 2a) and assisted suicide (subproject 2b); and (3) through verbal and nonverbal patient communication in palliative care settings (subproject 3)

Methods and analysis

- Subproject 1a: a cross-sectional study using validated and standardized questionnaires
- Subprojects 1b and 1c: a constructivist grounded theory method aiming at developing a novel theory from semi-structured interviews in both patients and nurses.
- Subproject 2a: a thematic analysis based on (i) audio-recordings of advance care planning encounters and (ii) follow-up semi-directive interviews with patients and their relatives
- Subproject 2b: a qualitative study based on thematic analysis of interviews with patients actively pursuing assisted suicide and one of their relatives.
- Subproject 3: a conversation analysis based on audio and video-recorded interactions in two settings: (i) palliative inpatient unit and (ii) advance care planning discussions.

Ethics and dissemination

The study project was approved by the Ethics Committees of the Canton of Vaud, Bern and Ticino (n° 2023-00088). In addition to participation in national and international conferences, each project will be the subject of two scientific publications in peer-reviewed journals. Additional publications will be realized according to result triangulations between projects. A symposium opened to professionals, patients and public will be organized in Switzerland at the end of the project.

Registration details

This work is supported by the Swiss National Science Foundation [grant number 10001G_207814 / 1].

Strengths and limitations

- We propose a set of multidisciplinary, complementary, and interlinked studies to explore altruism in palliative care in three different settings
- A complex concept such as patient altruism can only be addressed if several disciplines and methods are used
- Each subproject is conceived to explore a specific facet of patient altruism at the end of life and relies on a specific methodology of analysis corresponding to their research questions

For peer review only - http://bmjopen.bmj.com/site/about/guidelines.xhtml

- A joint phase of data and results triangulation between the subprojects will allow exchanges between teams on results and new interpretations of phenomena
- As a limitation, patients and public were not involved in the design of the study

For peer review only

For peer review only - http://bmjopen.bmj.com/site/about/guidelines.xhtml

INTRODUCTION

Palliative care challenges

In palliative care, the psychological domain is an essential component of patient care. Previous research in the field has mostly focused on improving the pharmacological and psychological treatments for the most frequent psychopathologies, i.e., anxiety, depression and adjustment disorders (1, 2). Less is known concerning the factors that foster psychological well-being, autonomy, and self-determination, thus improving quality of life. This approach, which has been termed "positive psychology" (3) parallels the resource-oriented, health-promoting approach known as salutogenesis. In recent years, palliative care patients have been considered as active participants in their own dying, but only concerning specific and well-defined aspects of the end of life, namely the so-called decisions concerning end-of-life (i.e. advance directives, withholding and withdrawing life-sustaining treatment, and requests for hastened death/assisted suicide where legally possible). In all other aspects concerning the last phase of life, patients are rather regarded as passive recipients of care, with only little opportunity to be perceived as "agents" who act, give, and contribute something by and of themselves. One possible way of fostering patient autonomy and well-being at the end of life is to consider the patient in line with the concept of "patient empowerment". While this paradigm is trending in many medical disciplines (4), it has had little impact in the field of palliative care so far, with the exception of those situations where decisions need to be made. It is precisely for this reason that we place the concept of patient altruism, as a form of patient agency, at the center of this project.

Nowadays, the concept of altruism is used in various disciplines such as psychology, philosophy, economics, sociology, and evolutionary biology (5, 6). Numerous authors tend to apply this concept to any prosocial behavior or action carried out on a voluntary basis and aiming to benefit the society at large, specific individuals or a group of individuals (5, 7-9). While altruistic behavior is primarily explained as an individual constitutive characteristic (10-13), this behavior has to be understood by considering its sources of motivation (benevolence (11), empathy (14), reward (15), norms (16), and anger (5, 17)). Social norms and interaction rules (social responsibility, group gain, reciprocity, negotiated rules) have also been identified as determinants of altruistic behaviors (18). To adequately address this multi-level concept (personality, motivations, social norms) an interdisciplinary approach appears necessary, which is also underscored by the different disciplines involved in this project (humanities, social sciences, medical sciences and nursing).

Although palliative care addresses all people suffering from a life-limiting illness, most palliative care patients who benefit from it are close to end of their life and are older of age. It is well established that older people report higher altruism levels than younger people (19). This is in agreement with life span developmental theories that have shown a change in motivation orientation during the last phase of existence, which promotes a sense of realization and meaning in life (20) . While meaning in life tends to increase with age (21, 22), the sources of meaning change: Sparrow et al. showed that, with increasing age, intrinsic values (such as authenticity, intimacy, spirituality but also altruism), are prioritized over extrinsic goals, such as achievement, competence, and power (23). Other authors mention a shift towards meaningful social goals focusing on others, especially close ones, after the recognition that time is becoming more limited as age increases (24). Vollhardt suggests that altruism may also be a consequence of suffering after adverse life events, such as a life-threatening illness (25, 26). In

For peer review only - http://bmjopen.bmj.com/site/about/guidelines.xhtml

line with these findings, Fegg et al. showed that palliative care patients consistently reported higher self-transcendent and altruistic values as compared to healthy adults (27). In addition, palliative care patients cite the social dimension as a source of meaning in life more often than the general population (28).

Since the fragility of patients at the end of life often reduces their capacity to undertake concrete altruistic actions by themselves, we need to broaden our understanding of altruism by also taking into account the attitudes and values at the origin of altruistic behaviors. In the theoretical model of basic human values developed by Schwartz, the values of "benevolence" and "universalism" ("self-transcendence") refer to concern for others' welfare and represent an internal motivation promoting social relations (29, 30). Since there is currently no clear definition or conceptual framework of altruism that has been applied to palliative care patients, we propose to define altruism in this context as: "a personal and intentional interest in improving the wellbeing and welfare of others – at an individual, group or societal level". By emphasizing the notion of "interest", we aim to include the different levels of manifestations of altruism that might be relevant to palliative care, including the (advance) decisions made by patients and the interactions upon which these decisions are based.

We therefore propose a set of multidisciplinary, complementary, and interlinked studies to explore altruism in palliative care in three different settings:

- The first level refers to patient altruism in the general context of palliative care:
  - We first aim to study to what extent altruism, considered in terms of prosocial behavior and self-transcendent value, is associated with important outcomes and indicators of palliative care (subproject 1a).
  - We then aim to explore (i) how altruism is defined and understood by the palliative care patients themselves (subproject 1b) and (ii) how altruism from patients is experienced by palliative care nurses, the professionals who are closest to patients in clinical practice, and how the expression of altruism by patients and families towards nurses influences professional meaning and fulfillment (subproject 1c).
- The second level considers how altruism might be expressed and realized through specific decisions between patients and their social environment by considering two specific decision-making contexts of high importance for palliative care: advance care planning (ACP, subproject 2a) and assisted suicide (subproject 2b). Both represent profound existential expressions of autonomy, which are closely related to the patients' values and attitudes.
- The third level concerns how altruism might be expressed in social interactions (both verbal or non-verbal), and how the interaction itself might have an altruistic dimension. We consider moments in which patients are involved in interaction by their own volition, and in which such interaction might bring a potential benefit to the other (subproject 3).

This project is anchored in an interdisciplinary effort bringing together the expertise of several researchers in palliative healthcare. They are therefore representative of the interdisciplinary nature of palliative care, as defined by the WHO (31). Mathieu Bernard, head of sub-projects 1a and 1b, is a psychologist; Phil Larkin, head of sub-project 1c, is a nurse; Ralf Jox, head of sub-project 2a, and Claudia Gamondi, head of sub-project 2b, are physicians, as Gian Domenico

For peer review only - http://bmjopen.bmj.com/site/about/guidelines.xhtml

Borasio, project coordinator. Finally, Anca-Cristina Sterie, head of sub-project 3, is a sociologist specialized in interactions in the medical setting.

The repercussions of this project could be manifold: first, it would allow designing and testing an altruism-based intervention for palliative care patients that could represent an important new step in the development of efficient, resource-oriented palliative care. Secondly, such interventions would have the potential to restore dignity and autonomy for patients in the last phase of life by allowing them, if they so wish, to assume a more active role. Thirdly, the expression of patient altruism towards family members and health care professionals could also profoundly affect the latter two: it could diminish their distress and ease their grieving, improve relationships and may be a role model for themselves to become more altruistic.

METHODS

Subproject 1a

A cross-sectional study using validated and standardized questionnaires will be conducted in both the French- and German-speaking parts of Switzerland (two University Hospitals and five palliative care centers), and two palliative care centers in the Italian-speaking part of Switzerland.

Inclusion criteria:

- 18 years or older
- treated by one of the palliative care teams
- <6 months life expectancy according to the treating physician
- medically stable state or when their state has improved

Exclusions criteria:

- evidence of psychiatric and cognitive symptoms that might significantly alter the decision-making capacity
- insufficient knowledge of the local language

Questionnaires and procedure

For the purpose of the study, we will use:

- The Prosocialness Scale for Adults (PSA) (32)
- The benevolence (four items) and universalism (six items) subscales of the 40-item Portrait Values Questionnaire (PVQ) (30)
- Quality of life will be measured with the McGill Quality of Life scale Revised version (MQOL-R) (33)
- Psychological distress will be measured with the Hospital Anxiety and Depression Scale (HADS) (34)
- Patient's feeling of being a burden to their caregivers will be assessed with the Self Perceived Burden Scale (SPBS) (35)
- Meaning in life will be assessed with the Meaning in Life Questionnaire (MLQ) (36)
- The will to live intensity will be measured with a single-item numerical rating scale (37)

These questionnaires were chosen because they measure dimensions that have been identified as psychological determinants of quality of life in palliative care. All new patients admitted in the palliative care centres participating in the study and who fulfil the inclusion and exclusion

For peer review only - http://bmjopen.bmj.com/site/about/guidelines.xhtml

criteria as assessed by the referring physician, will be asked by a research assistant to participate in the study as soon as an appropriate acute symptom control has been reached. Written informed consent will be obtained.

Validated translations or cross-cultural adaptations of the questionnaires will be used in face-to-face interviews. In addition, sociodemographic variables and medical data (principal diagnosis, co morbidities, performance status) will be obtained. Finally, questions using numerical rating scales (0-10) will assess the level of stress and the potential for personal development induced by the questionnaires. Such numerical rating scales will also be used to assess (i) to what extent altruism is an important concept in the end-of-life context, and (ii) to what extent patients feel frustration not to be able to express altruism towards others.

Data analysis strategy

Rates will be reported for the recruitment information. Descriptive statistics will be calculated for socio-demographic variables and all outcomes. Missing outcome data will be treated as mentioned in the scoring manuals. Distribution of each outcome measures will be analysed using normality tests and other indicators of distribution (box-plots, skewness and kurtosis estimations). Associations between outcome variables will be assessed using Pearson or Spearman correlations according to distributions. Univariate and multiple linear regressions will be performed in order to determine whether altruism can be considered as a significant explanatory variable (in addition to psychological distress, desire to live, feeling of being a burden and meaning in life) for quality of life. We will use an alpha threshold <.05 as a criterion for rejecting the null hypothesis. All models will be controlled for socio-demographic characteristics.

Sample size

Multiple linear regressions represent the crucial elements for assessing the number of participants to be included to ensure sufficient power. According to Howell (38), we aim to recruit 15 patients per explanatory variable, i.e. 120 patients with complete data in this study (50 in the French- and German-speaking parts and 20 in the Italian-speaking part, according to population representativeness). Estimating an exclusion rate of 60% and a refusal rate of 50% based on previous studies in the same context (39-41), 600 patients in total will need to be screened for study eligibility (250 in the French- and German-speaking parts and 100 in the Italian-speaking part).

Subproject 1b

Procedure and sample size

A subsample of participants from project 1a will be invited to participate in subproject 1b. Agreement to participate will be sought at the end of project 1a data collection. We estimate a maximum sample of 45 participants (approximately 15 per linguistic region), depending on data saturation (REF) and the need to explore items in greater or lesser depth. Therefore, the final sample may not be determined until the end of the study. We will aim to maximise the sample variation by ensuring diversity across gender, age, setting of care, illness type (e.g. cancer vs. non-cancer), and also by considering the score on the two questionnaires assessing altruism.

A constructivist grounded theory method will be adopted for project 1b and 1c (42). In this qualitative method the researcher inductively analyses individual and collective actions as well

For peer review only - http://bmjopen.bmj.com/site/about/guidelines.xhtml

as social and psychological processes, approaching the data with no preconceived ideas or hypotheses, accepting that there may be multiple perspectives and acknowledging the importance of their own place in constructing those perspectives with participants. Data collection and analysis are undertaken in parallel and decisions on sampling are revised as the project develops, a process referred to as 'theoretical sampling'.

An initial interview guide will be developed based on topics derived from data collected in subproject 1a and from research questions of subproject 1b. Semi-structured interviews will be conducted by a researcher trained in qualitative methodology and grounded theory data analysis, supported by qualitative research experts. The semi-structured approach enables the interviewer to vary the wording and sequence of the questions and pursue leads provided by the participants, while being respectful of the burden of interviewing a frail and potentially vulnerable population.

The interview guide will be developed by the research team, comprising native speakers in French, German, Italian and English. The team will meet after the first five interviews in each region to discuss emerging themes and agree if new directions in terms of revised questions are needed. Theoretical saturation will be determined first in each region and later agreed through consensus with the wider research team.

Data analysis

All interviews will be digitally recorded and transcribed in the language of origin. To account for language and cultural differences, interpretation and analysis of the data will be carried out by research collaborators in each region, trained in qualitative methodology and supported by the local research partners. Data will be entered into MAXQDA software to assist analysis.

In accordance with constructivist grounded theory methods, a broad pool of codes will be obtained during an open (initial) coding using interview data, notes and memos. In a second phase of axial (focused) coding, all individual codes will be sorted and resorted as concepts and common themes are developed (42). In the final step, (theoretical coding) overarching themes will be grouped into more refined categories and central concepts, again referring to wider data sources (notes, memos, etc.). Each coding step will be conducted in the individual language for the region and data shared through regular collaborative meetings to ensure accuracy and quality. An iterative discussion process will be engaged throughout the study, in order to reach a substantial agreement between researchers in terms of major themes and any cultural or regional difference identified. The final themes will be translated into English as the common language for the presentation of data and subjected to a second level inductive analysis and comparison with the host language for accuracy in terms of conceptual equivalence. The emerging theory will be co-constructed by the research team.

Subproject 1c

Procedure and sample size

We first begin with a conceptual analysis of altruism to determine its antecedents, attributes and relationships with palliative care nursing. Conceptual analyses are of particular benefit where a concept requires refinement within a specific discipline or context (43). This will also inform the development of the interview guide and the outcome can be refined during interviews with the nursing participants.

For peer review only - http://bmjopen.bmj.com/site/about/guidelines.xhtml

Following the constructivist grounded theory principles outlined in subproject 1b, 30 semi-structured in-depth interviews with nurses working in palliative care nursing practice will be undertaken. Participants will be accessed through the same University Hospitals and palliative care centres collaborating for subprojects 1a and 1b (French, German and Italian). Interviews will provide concrete examples of the nurses' experience of altruism in practice and address the meaning and impact of altruism for the palliative care nurse practitioner.

Inclusion criteria for nurses:

- Working at specialist or generalist level within an agreed palliative care setting
- Having worked in a full-time or part-time capacity for at least 6 months to be able to reflect and discuss their professional experience and offer concrete examples from practice.
- Willing to be interviewed and recorded
- Able to converse in either French, German, Italian or English

Since the division between specialist and generalist palliative care nursing roles is ill-defined, interviews with a broader nursing sample will help to understand the broader facilitators and barriers to altruism and to seek ulterior expressions and meanings of altruism within a wider professional nursing cohort. Ten interviews per linguistic region are planned.

Data analysis

The process of data analysis described in subproject 1b will also be applied to subproject 1c for symmetry and completeness of data across subprojects 1b and 1c. It will then afford opportunity to consider both data sets for similarities and disparities in the understanding and expression of altruism.

Subproject 2a

In order to address the aims of subproject 2a, a qualitative design will be used with three types of data; (i) audio or video-recorded routine ACP discussions between a facilitator, a patient and their relative(s) if applicable; (ii) semi-structured follow-up interviews of the persons having participated in these discussions.

Inclusion criteria for patients

- 18 years or older
- receiving general or specialized palliative care

Inclusion criteria for relatives

- being nominated and recruited by the patient
- 18 years or older
- capable to participate to an interview in French or German

Inclusion criteria for ACP facilitators

- acting as facilitator of ACP discussions with the patient (and his or her relatives)
- being trained as ACP facilitator

Exclusion criteria for patients and relatives

- evidence of psychiatric or cognitive symptoms significantly altering the decision-making capacity

For peer review only - http://bmjopen.bmj.com/site/about/guidelines.xhtml

Procedure and sample size

We will follow a sampling strategy combining cluster sampling and convenience sampling. The cluster sampling will be oriented towards variation regarding gender, age groups and health status of the patient participants (oncological, neurological and cardiorespiratory illnesses). The convenience part of the sampling relates to the fact that we will recruit in structured and professionally facilitated ACP activities in the University Hospitals of both the French and German parts of Switzerland, and in two palliative care centers of the Italian part of Switzerland. It is planned to conduct 9-12 qualitative interviews with ACP facilitators (3-4 per language region), and to register 24 audio-recorded routine ACP discussions (8 in each language area), as well as 24 follow-up interviews with patients and 24 follow-up interviews with relatives having participated in the ACP discussions.

Data collection

The ACP discussions will be audio or video recorded. Audio-recordings will be transcribed verbatim. The transcripts will then be coded by the investigator and analyzed by the researcher.

The semi-structured interviews will be conducted face-to-face with the patients, their relatives, and the ACP facilitators in the participants' native language, audio-taped and integrally transcribed anonymizing all personal details. The interview grid will be constructed following both an inductive and a deductive approach. The initial grid will be deducted from the research team's experience in the topic and the literature. Five pilot interviews will be carried out to test the interview design. Whether the pilot interviews will be included in the data set will be decided depending on the evaluation of their transcripts.

Video and audio data will serve for further analysis in subproject 3.

Data analysis

For data analysis, please see below at project 2b

Subproject 2b

To address the aims of subproject 2b, a qualitative study will be used based on semi-structured interviews with patients and one of their relatives regarding the patient's expression of their wish to die by assisted suicide. Participants will be recruited in the French, German, and Italian-speaking parts of Switzerland.

Inclusion criteria for patients

- expression of wish to die by assisted suicide
- being registered to a right-to-die association in Switzerland
- 18 years or older
- capable of participating in an interview in French or Italian or German

Inclusion criteria for relatives

- being nominated and recruited by the patient
- having been informed by the patient of their intention to obtain assistance in suicide
- 18 years or older
- capable of participating in an interview in French or

For peer review only - http://bmjopen.bmj.com/site/about/guidelines.xhtml

Exclusion criteria for patients and relatives

- evidence of psychiatric symptoms or cognitive impairment that might significantly alter the decision-making capacity.

Procedure and sample size

Recruitment will take place through different sources; right to die associations and providers operating in the field of specialised palliative care. Snowball sampling will be also used. Ten patients and 10 relatives from the French-speaking part of Switzerland, 10 patients and 10 relatives from the German-speaking part of Switzerland, and 5 patients and 5 relatives from the Italian-speaking part of Switzerland will be recruited.

We intend to disseminate information about the study through two main channels:

- Specialized palliative care centers of the University Hospitals in the French and German parts of Switzerland, and two palliative care centers of the Italian part of Switzerland. Recruitment will be non-systematic: health professionals within these services will inform the study investigator when a patient meets the criteria

Right-to-die societies that will inform their members actively pursuing assisted suicide decision about the study

- Interviews will be conducted face-to-face with both the patients and their relatives. The interview grid will be constructed following the same procedure as described for subproject 2a.

Data analysis for sub-projects 2a and 2b

The approach for data analysis is the same for sub-projects 2a and 2b. To account for language and cultural differences, each coding step will be conducted in the regional language in which data was collected and data will then be shared through regular collaborative meetings. To ensure accuracy and quality in data generated, results will be translated into English and merged for a final transversal analysis.

The analysis will follow an inductive paradigm derived from thematic analysis (44). The interviews will be fragmented into significant text units, to which codes, or designations able to synthetically account for their content, will be assigned. The identified codes will be linked and grouped into larger categories to define more abstract concepts around which to organize the various arguments. 25% of the material will be double coded independently by another researcher affiliated to the project, to allow for parallel coding. For subproject 2a, each data subset (ACP encounters and follow-up interviews) will be analyzed individually as well as jointly. These operations will be made with the support of the specialized software for qualitative data analysis MAXQDA.

Our analysis will also account for the fact that the decisions involved in these subsets of data (ACP and assisted suicide) have important ethical implications. We will therefore undertake further conceptual analysis inspired by methods from analytical philosophy and the frameworks of the principles of biomedical ethics (45) and of care ethics (46). Normative criteria will be identified to ethically evaluate altruistic acts at the end of life, based on the principles of biomedical ethics (45). Finally, we will present practical recommendations that promote the co-existence of patient autonomy and patient altruism.

For peer review only - http://bmjopen.bmj.com/site/about/guidelines.xhtml

Subproject 3

Procedure and sample size

This subproject relies on natural data: audio and/or video recorded naturally occurring interactions, taking place spontaneously, i.e., not generated for the purpose of this study. Data will be collected in two settings in the French part of Switzerland

1. Advance Care Planning encounters

We will use data recorded for the sub-project 2a.

2. Hospital palliative care units

We will record interactions taking place during 40 patient hospitalizations in three palliative centres in French Switzerland. This concerns activities taking place in the patient's room, involving the patient, their visitors, and palliative care professionals participating to the study. The term 'activity' will be used in the broad sense, comprising verbal and non-verbal exchanges and acts of care. Participants will be offered the option to decide, in advance, what type of activities they agree to be recorded in; consent will be reconfirmed prior to each recording. Participants will be offered the option of consenting to audio and video, or just to audio recording.

Inclusion criteria for patients

- hospitalized in one of three palliative care centres
- medically stable or when their state has improved

Exclusion criteria for patients

- imminence of death according to the referent physician
- impaired decision-making capacity
- presence of psychological/psychiatric problems due to which participating (being recorded) might harm the patient

Inclusion criteria for health care professionals

- All health and allied health professionals affiliated to the palliative care unit

Inclusion criteria for visitors and relatives

- Participation is open to all visitors of patients participating to the study

Data analysis

Analysis will be guided by the conversation analysis (CA) approach. CA resides in a finely-grained analysis of recorded data, focusing on how participants interact in order to accomplish ordinary as well as interactionally challenging tasks (47). While CA is an inductive approach, its use is regulated by a well-defined and stepwise process:

- The first-stage analysis is done as an "initial noticing", in order to identify details of talk ("phenomena") that are interesting from a research point of view but also recurrent throughout the data (48).

For peer review only - http://bmjopen.bmj.com/site/about/guidelines.xhtml

- Secondly, the researcher starts an exhaustive search throughout all the instances in which the phenomena occur and gathers them in data sets. Sequences of talk in which the phenomena are identified are transcribed according to a CA convention system designed for linguistic and multi-modal transcriptions, which takes into account aspects of speech delivery and representation of activities parallel to talk (eye gaze, laughing, motions) (49-51).
- Thirdly, the essential part of the analysis involves describing the phenomena in terms of sequential location (where it appears, why, what it generates) and content. The analysis will particularly draw on concepts of 'affiliation' (52), 'benefactors and beneficiaries' (53) and empathic communication (54) developed in CA and in relation to the field of palliative care (55-57).

Data collected in the first setting (ACP conversations) will be monitored for how patients participate to the discussion of medical decisions, especially how patients may be "pro-social" by orienting themselves towards others when talking e.g. about death-related topics, without being required to do so. This is in keeping with our definition of altruism.

Data collected in the second setting (hospital palliative care) will be monitored for when and how patients might interact (verbally or non-verbally) without being prompted, and about what. The focus will be on localizing and analyzing sequences of interaction in which palliative patients interact by their own volition. These instances will be investigated focusing on whether the patient's involvement might be identified as being done for the benefit of the other person (e.g. make a compliment, participate to an act of care or an exchange without being asked/required to).

Patient and Public Involvement

No patients or members of the public were involved in the design of the study for any of the sub-projects.

ETHICS AND DISSEMINATION

The study project was approved by the Ethics Committees of the Canton of Vaud, Bern and Ticino (n°2023-00088). Each project will be the subject of two scientific publications in peer-reviewed journals. Additional publications will be realized according to result triangulations between projects. The results will also be presented at international and national conferences. Finally, a symposium opened to professionals, patients and public will be organized in Switzerland at the end of the project to present all the results.

REFERENCES

1. Mitchell AJ, Chan M, Bhatti H, Halton M, Grassi L, Johansen C, et al. Prevalence of depression, anxiety, and adjustment disorder in oncological, haematological, and palliative-care settings: a meta-analysis of 94 interview-based studies. The Lancet Oncology. 2011;12(2):160-74.
2. Parpa E, Tsilika E, Gennimata V, Mystakidou K. Elderly cancer patients' psychopathology: A systematic review Aging and mental health. Archives of Gerontology and Geriatrics. 2015;60(1):9-15.
3. Seligman, Csikszentmihalyi. Positive psychology. An introduction. Am Psychol. 2000;55(1):5-14.

For peer review only - http://bmjopen.bmj.com/site/about/guidelines.xhtml

4. Pekonen A, Eloranta S, Stolt M, Virolainen P, Leino-Kilpi H. Measuring patient empowerment - A systematic review. Patient Educ Couns. 2020;103(4):777-87.
5. Fehr E, Gächter S. Altruistic punishment in humans. Nature. 2002;415(6868):137-40.
6. Sonne JWH, Gash DM. Psychopathy to Altruism: Neurobiology of the Selfish-Selfless Spectrum. Frontiers in psychology. 2018;9:575.
7. FitzPatrick WJ. Human altruism, evolution and moral philosophy. Royal Society open science. 2017;4(8):170441.
8. Warneken F, Tomasello M. The roots of human altruism. British journal of psychology (London, England : 1953). 2009;100(Pt 3):455-71.
9. West SA, Gardner A, Griffin AS. Altruism. Current biology : CB. 2006;16(13):R482-3.
10. DeYoung CG, Quilty LC, Peterson JB. Between facets and domains: 10 aspects of the Big Five. Journal of personality and social psychology. 2007;93(5):880-96.
11. Hubbard J, Harbaugh WT, Srivastava S, Degras D, Mayr U. A general benevolence dimension that links neural, psychological, economic, and life-span data on altruistic tendencies. Journal of experimental psychology General. 2016;145(10):1351-8.
12. McCrae RR, John OP. An introduction to the five-factor model and its applications. Journal of personality. 1992;60(2):175-215.
13. Szuster A. Crucial Dimensions of Human Altruism. Affective vs. Conceptual Factors Leading to Helping or Reinforcing Others. Frontiers in psychology. 2016;7:519.
14. Batson CD, Batson JG, Slingsby JK, Harrell KL, Peekna HM, Todd RM. Empathic joy and the empathy-altruism hypothesis. Journal of personality and social psychology. 1991;61(3):413-26.
15. Carlo G, Hausmann A, Christiansen S, Randall BA. Sociocognitive and behavioral correlates of a measure of prosocial tendencies for adolescents. The Journal of Early Adolescence. 2003;23(1):107-34.
16. Chen H, Zeng Z, Ma J. The source of punishment matters: Third-party punishment restrains observers from selfish behaviors better than does second-party punishment by shaping norm perceptions. PloS one. 2020;15(3):e0229510.
17. Mussweiler T, Ockenfels A. Similarity increases altruistic punishment in humans. Proceedings of the National Academy of Sciences of the United States of America. 2013;110(48):19318-23.
18. Cropanzano R, Mitchell MS. Social exchange theory: An interdisciplinary review. [References]. Journal of Management. 2005;31(6):874-900.
19. Sparrow EP, Swirsky LT, Kudus F, Spaniol J. Aging and altruism: A meta-analysis. Psychology and aging. 2021;36(1):49-56.
20. Dunkel CS, Harbke C. A review of measures of Erikson's stages of psychosocial development: Evidence for a general factor. Journal of Adult Development. 2017;24(1):58-76.
21. Reker GT, Wong PTR. Personal meaning in life and psychosocial adaptation in the later years. [References]. In: Wong PTP, editor. The human quest for meaning: Theories, research, and applications. 2nd ed. New York, NY, US: Routledge/Taylor & Francis Group; US.; 2012. p. 433-56.
22. Reker GT, Woo LC. Personal meaning orientations and psychosocial adaptation in older adults. [References]: SAGE Open. Vol.1(1), 2011, pp. 1-10.; 2011.
23. Sparrow EP, Spaniol J. Aging and altruism in intertemporal choice. Psychology and aging. 2018;33(2):315-24.
24. Carstensen LL, Fung HH, Charles ST. Socioemotional selectivity theory and the regulation of emotion in the second half of life. [References]. Motivation and Emotion. 2003;27(2):103-23.
25. Staub E, Vollhardt J. Altruism born of suffering: the roots of caring and helping after victimization and other trauma. The American journal of orthopsychiatry. 2008;78(3):267-80.
26. Vollhardt JR. Altruism born of suffering and prosocial behavior following adverse life events: A review and conceptualization. [References]. Social Justice Research. 2009;22(1):53-97.
27. Fegg MJ, Wasner M, Neudert C, Borasio GD. Personal values and individual quality of life in palliative care patients. J Pain Symptom Manage. 2005;30(2):154-9.
28. Bernard M, Berchtold A, Strasser F, Gamondi C, Borasio GD. Meaning in life and quality of life: palliative care patients versus the general population. BMJ Support Palliat Care. 2020.

For peer review only - http://bmjopen.bmj.com/site/about/guidelines.xhtml

29. Sagiv L, Roccas S, Cieciuch J, Schwartz SH. Personal values in human life. Nature human behaviour. 2017;1(9):630-9.
30. Schwartz SH, Cieciuch J. Measuring the Refined Theory of Individual Values in 49 Cultural Groups: Psychometrics of the Revised Portrait Value Questionnaire. Assessment. 2021:1073191121998760.
31. World Health Organisation. Global atlas of palliative care at the end of life 2014 [Available from: http://www.who.int/cancer/palliative/definition/en/.
32. Caprara GV, Steca P, Zelli A, Capanna C. A new scale for measuring adults' prosocialness. European Journal of Psychological Assessment. 2005;21(2):77-89.
33. Cohen SR, Sawatzky R, Russell LB, Shahidi J, Heyland DK, Gadermann AM. Measuring the quality of life of people at the end of life: The McGill Quality of Life Questionnaire-Revised. Palliat Med. 2017;31(2):120-9.
34. Zigmond AS, Snaith RP. The hospital anxiety and depression scale. Acta psychiatrica Scandinavica. 1983;67(6):361-70.
35. Cousineau N, McDowell I, Hotz S, Hébert P. Measuring chronic patients' feelings of being a burden to their caregivers: development and preliminary validation of a scale. Medical care. 2003;41(1):110-8.
36. Naghiyaee M, Bahmani B, Asgari A. The Psychometric Properties of the Meaning in Life Questionnaire (MLQ) in Patients with Life-Threatening Illnesses. TheScientificWorldJournal. 2020;2020:8361602.
37. Bornet MA, Bernard M, Jaques C, Rubli Truchard E, Borasio GD, Jox RJ. Assessing the Will to Live: A Scoping Review. J Pain Symptom Manage. 2021;61(4):845-57.e18.
38. Howell CA. Statistical methods for psychology. Seventh edition ed. Belmont, CA, USA: Cengage Wadsworth; 2017.
39. Althaus B, Borasio GD, Bernard M. Gratitude at the End of Life: A Promising Lead for Palliative Care. J Palliat Med. 2018;21(11):1566-72.
40. Bernard M, Strasser F, Gamondi C, Braunschweig G, Forster M, Kaspers-Elekes K, et al. Relationship Between Spirituality, Meaning in Life, Psychological Distress, Wish for Hastened Death, and Their Influence on Quality of Life in Palliative Care Patients. J Pain Symptom Manage. 2017;54(4):514-22.
41. Brenner KO, Rosenberg LB, Cramer MA, Jacobsen JC, Applebaum AJ, Block SD, et al. Exploring the Psychological Aspects of Palliative Care: Lessons Learned from an Interdisciplinary Seminar of Experts. J Palliat Med. 2021;24(9):1274-9.
42. Charmaz K. Constructing grounded theory. 2nd ed. Thousand Oaks, CA, US: Sage publications; 2014.
43. Sharifi N, Adib-Hajbaghery M, Najafi M. Cultural competence in nursing: A concept analysis. Int J Nurs Stud. 2019;99:103386.
44. Braun V, Clarke V. What can "thematic analysis" offer health and wellbeing researchers? Int J Qual Stud Health Well-being. 2014;9:26152.
45. Beauchamp T, Childress J. Principles of Biomedical Ethics: Marking Its Fortieth Anniversary. Am J Bioeth. 2019;19(11):9-12.
46. Gilligan C. In A Different Voice. Cambridge: Harvard University Press; 1982.
47. Schegloff EA. Sequence organization in interaction: A primer in conversation analysis I: Cambridge university press; 2007.
48. Schegloff EA. Confirming allusions: Toward an empirical account of action. American journal of sociology. 1996;102(1):161-216.
49. Jefferson G. Glossary of transcript symbols with an introduction. In: Lerner GH, editor. Conversation Analysis: Studies from the first generation: John Benjamins; 2004. p. 13-34.
50. Parry R. Video-based conversation analysis. Sage handbook of qualitative methods in health research. London: Sage; 2010. p. 373-96.
51. Rossano F. Gaze in Conversation. In: Sidnell J, Stivers T, editors. The handbook of conversation analysis: Blackwell Publishing; 2013. p. 308-29.

For peer review only - http://bmjopen.bmj.com/site/about/guidelines.xhtml

52. Steensig J. Conversation analysis and affiliation and alignment. The encyclopedia of applied linguistics: Wiley; 2012. p. 1-6.

53. Clayman SE, Heritage J. Benefactors and beneficiaries: Benefactive Status and Stance in the Management of Offers and Requests. In: Couper-Kuhlen E, Drew P, editors. Requesting in social interaction. Amsterdam: John Benjamins; 2014. p. 55-86.

54. Heritage J, Maynard DW. Communication in medical care: Interaction between primary care physicians and patients: Cambridge University Press; 2006.

55. Gramling D, Gramling R. Palliative care conversations: clinical and applied linguistic perspectives: Walter de Gruyter GmbH & Co KG; 2019.

56. Jenkins L, Parry R, Pino MJAL. Providing opportunities for patients to say more about their pain without overtly asking: a conversation analysis of doctors repeating patient answers in palliative care pain assessment. Applied Linguistics. 2021:1-24.

57. Land V, Parry R, Pino M, Jenkins L, Feathers L, Faull C. Addressing possible problems with patients' expectations, plans and decisions for the future: One strategy used by experienced clinicians in advance care planning conversations. Patient Education and Counseling. 2019;102(4):670-9.

AUTHORS' CONTRIBTIONS

MB and GDB designed the study protocol. MB developed the study design and method of subproject 1a; MB and PL developed the study design and method OF subproject 1b; PL developed the study design and method of subproject 1c; RJ developed the study design and method of subproject 2a; CG developed the study design and method of subproject 2b; AS developed the study design and method of subproject 3.

FUNDING STATEMENT

This work is supported by the Swiss National Science Foundation [grant number 10001G_207814 / 1]. The study is funded for a three years period from April 2023 to March 2026.

COMPETING INTERESTS STATEMENT

The authors declared no potential conflicts of interest with respect to the research, authorship, and/or publication of this article.

For peer review only

For peer review only - http://bmjopen.bmj.com/site/about/guidelines.xhtml
