# Supplementary material for: Interdisciplinary research approach based on a mixed-methods design to explore patient altruism at the end of life: a study protocol
Source: BMJ Open. 2024 May 10;14(5):e085632. doi: 10.1136/bmjopen-2024-085632 (PMC11097832; doi:10.1136/bmjopen-2024-085632)
Supplement: Reviewer comments [file bmjopen-2024-085632.reviewer_comments.pdf]

PEER REVIEW HISTORY

BMJ Open publishes all reviews undertaken for accepted manuscripts. Reviewers are asked to complete a checklist review form (**http://bmjopen.bmj.com/site/about/resources/checklist.pdf**) and are provided with free text boxes to elaborate on their assessment. These free text comments are reproduced below.

ARTICLE DETAILS

**TITLE (PROVISIONAL)**	An interdisciplinary research approach based on a mixed methods design to explore patient altruism at the end of life: a study protocol
**AUTHORS**	Bernard, Mathieu; Gamondi, Claudia; Sterie, Anca-Cristina; Larkin, Philip J; Jox, Ralf; Borasio, Gian Domenico

This article was not externally reviewed at BMJ Open. Protocols that have been independently assessed prior to submission to BMJ Open are usually fast-tracked to publication on the grounds that further substantial changes will not be possible. This independent assessment will usually be external, independent review for both a substantial grant award from a non-commercial or government funder and ethics approval.
